# Bionic Artificial Leaves Based on AIE‐Active Supramolecular Hydrogel for Efficient Photocatalysis

**DOI:** 10.1002/advs.202504993

**Published:** 2025-05-08

**Authors:** Rongbo Zhang, Xueqi Tian, Minzan Zuo, Tao Zhang, Srikala Pangannaya, Xiao‐Yu Hu

**Affiliations:** ^1^ College of Materials Science and Technology Nanjing University of Aeronautics and Astronautics Nanjing 211106 China; ^2^ College of Chemistry and Materials Jiangxi Normal University Nanchang 330022 China; ^3^ Department of Chemistry, School of Humanities and Sciences Gokaraju Rangaraju Institute of Engineering and Technology Bachupally Hyderabad Telangana 500090 India; ^4^ Qinghai Provincial Key Laboratory of Tibetan Medicine Research Northwest Institute of Plateau Biology Chinese Academy of Sciences Xining 810008 China

**Keywords:** aggregation‐induced emission, energy transfer, light‐harvesting system, photocatalysis, supramolecular hydrogel

## Abstract

A novel hydrogel‐based biomimetic artificial leaf is fabricated by integrating host‐guest interactions with covalent bonding. Specifically, a water‐soluble tetraphenylethylene‐embedded pillar[5]arene (***m*‐TPEWP5**), which exhibits aggregation‐induced emission (AIE) property, is synthesized as the host molecule. An amphiphilic guest **G** is introduced to form a stable complex (**HGSM**) via non‐covalent interactions. Subsequent copolymerization of **HGSM** with gelatin methacryloyl (**GelMA**) yields a hydrogel network (**HGGelMA**), which not only exhibits AIE characteristics but also enables encapsulation of the acceptor eosin Y (ESY), thereby resulting in the construction of an artificial light‐harvesting system **HGGelMA**⊃ESY that serves as a biomimetic leaf. To emulate natural photosynthesis more closely and optimize the utilization of the collected energy, two organic reactions are performed within this artificial leaf: dehalogenation of bromoacetophenone derivatives and coupling of benzylamine. These reactions demonstrate remarkable catalytic activity and recycling ability during the photocatalytic process.

## Graphical abstract

A novel bionic artificial leaf is developed by integrating host‐guest interactions with covalent bonding. *Meso*‐functionalized pillararene (***m*‐TPEWP5**) formed a stable host‐guest complex **HGSM** with guest **G**, which is copolymerized with gelatin methacryloyl (**GelMA**) to form a hydrogel **HGGelMA** for encapsulating eosin Y (ESY). The resulting **HGGelMA**⊃ESY system serves as an efficient artificial light‐harvesting apparatus, catalyzing the dehalogenation of bromoacetophenone derivatives and the coupling of benzylamine derivatives.

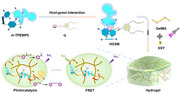

## 1 Introduction

In response to the growing global emphasis on sustainable development and environmental preservation, renewable energy technologies have emerged as a crucial strategy for addressing the escalating energy crisis. Among these, solar energy has garnered significant attention from the scientific community as a clean and renewable power source.^[^ ^1^ ^]^ Photosynthesis, an exceptional process that sustains green plants and algae, exemplifies the efficient conversion of solar energy into chemical energy for storage.^[^ ^2^ ^]^ This process begins with the absorption of sunlight by antenna pigment complexes located within chloroplasts, followed by a highly synergistic mechanism that effectively transfers excitation energy to the energy receptors of the reaction centers.^[^ ^3^ ^]^ Inspired by photosynthesis, numerous researchers have pursued innovations in developing efficient donor‐acceptor systems based on Förster resonance energy transfer (FRET) processes, aiming to construct effective light‐harvesting systems (LHSs).^[^ ^4^ ^]^ Advances in supramolecular chemistry offer innovative tools and methodologies for designing LHSs,^[^ ^5^ ^]^ primarily because of their emphasis on noncovalent intermolecular interactions, which enable the assembly of complex structures with specific functions.^[^ ^6^ ^]^ However, current research on supramolecular systems in the context of LHSs predominantly focuses on light harvesting, which does not fully exploit the captured energy.^[^ ^7^ ^]^

The rapid advancement in photocatalytic technology offers a promising solution to address the existing limitation, as photocatalysts can facilitate chemical reactions under illumination and directly convert solar energy into chemical energy.^[^ ^8^ ^]^ By integrating supramolecular systems with photocatalytic technology, it is feasible to construct more efficient artificial light‐harvesting and photocatalytic systems that synergistically combine the functions of light capture and energy utilization.^[^ ^9^ ^]^ For example, LHSs capable of energy transfer have been constructed using supramolecular strategies and successfully applied to catalyze various reactions, including dehalogenation,^[^ ^10^ ^]^ cross‐dehydrogenative coupling,^[^ ^11^ ^]^ hydrogen evolution^[^ ^12^ ^]^ and oxidation.^[^ ^4^, ^13^ ^]^ These LHSs mimic the natural process of photosynthesis, promoting efficient energy transfer and the catalysis of chemical transformations. However, most existing LHSs for photocatalysis are constrained by solubility issues and are typically confined to homogeneous systems, complicating their recyclability.^[^ ^14^ ^]^ Therefore, developing a heterogeneous LHS suitable for aqueous media while maintaining recyclability is crucial for the efficient utilization of light energy and the advancement of photocatalytic technology.^[^ ^15^ ^]^ To address this challenge, our research group has pioneered an innovative approach to construct heterogeneous LHS by leveraging ***m*‐TPE Di‐EtP5** as the host. This system forms a supramolecular complex with a dinitrile derivative guest, resulting in precipitation from solution and facilitating efficient catalysis of dehalogenation reactions. Nevertheless, the reaction scope of this system remains restricted.^[^ ^16^ ^]^

In this work, we developed a novel LHS by integrating the FRET process into a hydrogel matrix, with the objective of catalyzing diverse reactions while ensuring recyclability. Specifically, we synthesized a *meso*‐functionalized water‐soluble pillar[5]arene derivative (***m*‐TPEWP5**), which exhibits aggregation‐induced emission (AIE) properties, as the host molecule. For the guest molecule, we designed a sulfonate‐terminated alkyl chain with a flexible glycol chain‐linked acrylate group at the opposite end. This guest molecule can bind to ***m*‐TPEWP5** via host‐guest interaction, forming a high‐performance supramolecular complex (HGSM). Subsequently, a hydrogel material **HGGelMA** was obtained through a covalent cross‐linking strategy involving the copolymerization of gelatin methacryloyl (**GelMA**) with **HGSM**. In this system, **HGSM** serves as an energy donor, encapsulating the energy acceptor eosin Y (ESY) to construct of an efficient LHS (**HGGelMA**⊃ESY), which can mimic the function of a biomimetic artificial leaf. The resulting artificial leaves significantly enhance the photocatalytic efficiency in dehalogenation reactions and act as type II photosensitizer, effectively generating singlet oxygen (^1^O_2_) to facilitate benzylamine coupling reaction. Both of the aforementioned photocatalytic reactions demonstrate that the artificial leaves **HGGelMA**⊃ESY possess excellent catalytic activity and recycling stability, highlighting their promising potential for applications in photocatalysis.

## 2 Results and Discussion

**Scheme 1:**
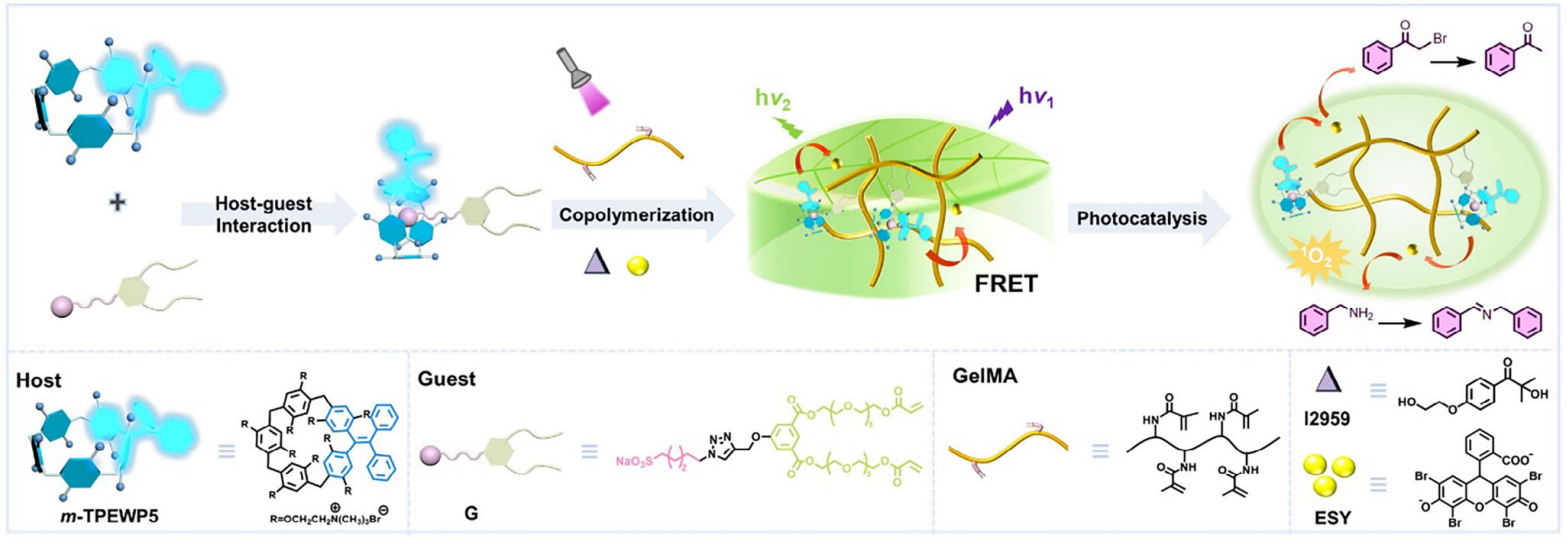
Schematic illustration of artificial leaves and their photocatalytic processes.

Initially, building upon our earlier research, the AIE‐active pillar[5]arene, designated as ***m*‐TPEWP5**, was synthesized through methylene bridge modifications at the *meso*‐position (Scheme S1, Supporting Information).^[^ ^17^ ^]^ Concurrently, an amphiphilic guest molecule **G** was designed and synthesized, with detailed procedures provided in the ESI (Scheme S2 and Figures S1–S7, Supporting Information). As shown in **Scheme** **1**, guest molecule **G** features two acrylate‐modified glycol chains, each terminating in a double bond that can undergo photoinitiated polymerization. The other end of **G** consists of an alkyl chain terminated with a sodium sulfonate group, which not only enhances the water solubility of the guest but also facilitates its electrostatic interaction with the host. Based on our previous findings,^[^ ^17^, ^18^ ^]^ the sulfonate‐terminated alkyl chain of guest **G** can enter the cavity of ***m*‐TPEWP5** through both electrostatic and hydrophobic interactions, confirming the formation of a 1:1 stoichiometric assembly between ***m*‐TPEWP5** and **G**.

**Figure 1:**
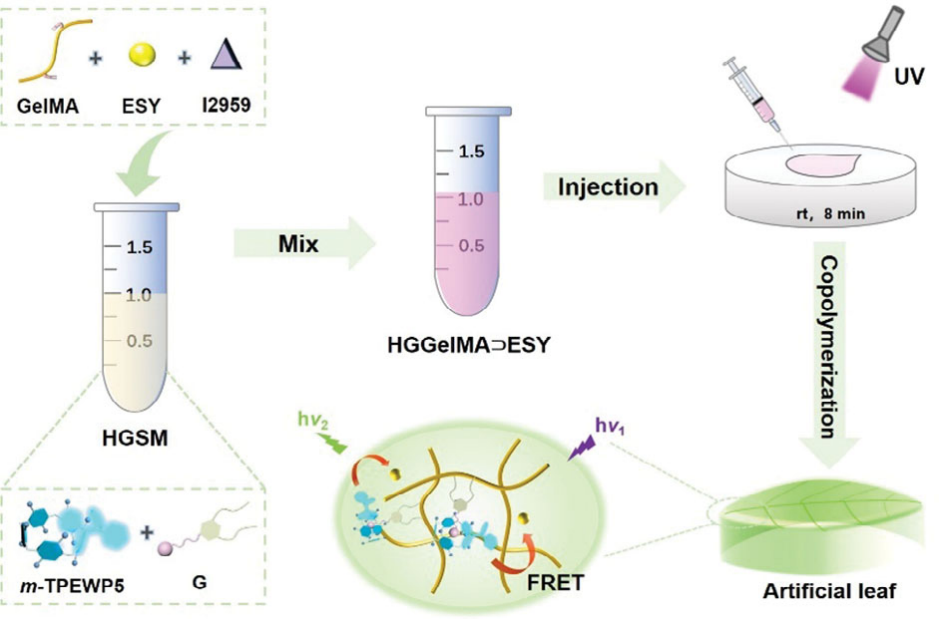
Schematic diagram illustrating the preparation of hydrogel **HGGelMA**⊃ESY using the host molecule ***m*‐TPEWP5**, the guest molecule **G,** and **GelMA** along with ESY and I2959. h*v*_1_ denotes the excitation wavelength of 365 nm, while h*v*_2_ indicates the emission wavelength of 565 nm.

Due to the limited solubility of the guest molecule, we utilized **G_M_** as a model guest to investigate its host‐guest interaction with ***m*‐TPEWP5** by ^1^H NMR spectroscopy in D_2_O. As illustrated by the NMR signals (Figure S8, Supporting Information), the addition of **G_M_** (1.0 equiv.) to ***m*‐TPEWP5** in solution resulted in an upfield shift of protons assigned to H_a‐f_ in **G_M_**. This observation clearly indicates that the alkyl chain was partially incorporated into the electron‐rich cavity of ***m*‐TPEWP5**. Subsequently, 1.0 equiv. of **G** was introduced into an aqueous solution of ***m*‐TPEWP5** and mixed thoroughly to form the **HGSM** solution. **GelMA** was synthesized by modifying gelatin with methacrylic anhydride. Specifically, 2.0 g of gelatin was dissolved in DPBS (Dulbecco's Phosphate‐Buffered Saline) at 60 °C. Once fully dissolved, 1.2 mL of methacrylic anhydride was added, and the reaction proceeded at 50 °C for 4 h. The reaction was then quenched by adding DPBS solution at 40 °C, followed by dialysis of the crude product in deionized water at 40 °C for one week. Finally, the dialyzed product was freeze‐dried to yield a white, foamy **GelMA**. ^1^H NMR spectral analysis (Figure S9, Supporting Information) confirmed that the degree of methacryloyl modification in **GelMA** was ≈67%.

**Figure 2:**
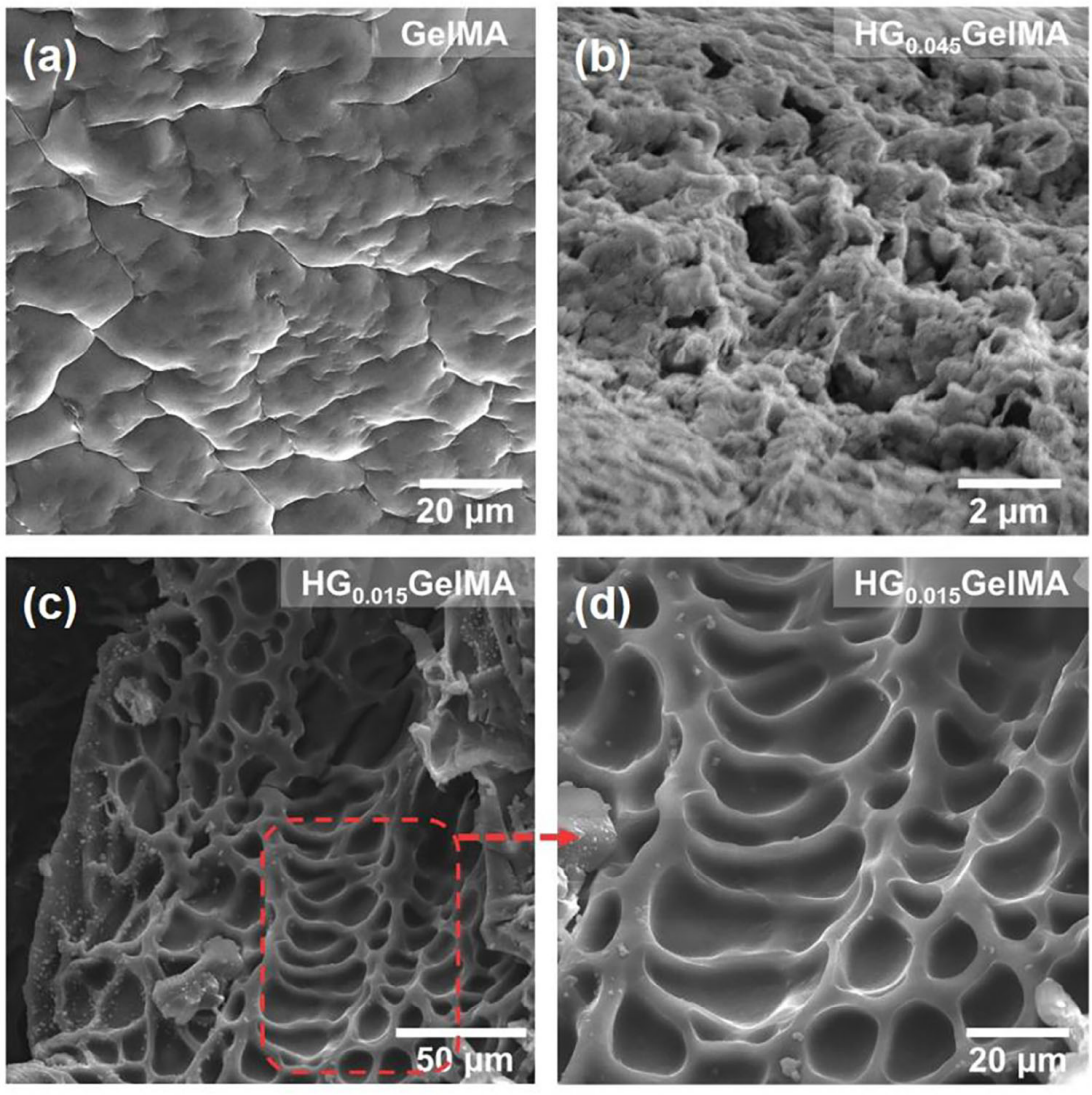
SEM images of a) **GelMA**, b) **HG_0.045_GelMA**, and c,d), **HG_0.015_GelMA**.

**HGSM** and **GelMA** were photopolymerized in aqueous solution ([**HGSM**]/[**GelMA**] = 1:3, [**HGSM**] = 0.015 mol L^−1^) under UV light irradiation (32 W) with 2‐hydroxy‐4‐(2‐hydroxyethoxy)benzophenone (I2959) as the photoinitiator. After 8 min of irradiation, **HGSM** and **GelMA** can be covalently cross‐linked to form supramolecular hydrogel **HGGelMA** (Figure 1). To investigate the internal structure of **HGGelMA**, the truncated surface of the lyophilized supramolecular hydrogel was examined by scanning electron microscope (SEM). SEM images revealed that pure **GelMA** exhibits a smooth surface with a tightly packed lamellar structure (**Figure** **2**a). In contrast, the incorporation of **HGSM** altered the cross‐linking structure of **GelMA**, resulting in a porous microstructure within **HGGelMA**. Notably, increasing the concentration of **HGSM** ([**HGSM**] = 0.045 mol L^−1^) led to denser cross‐linking, which reduced the size of internal micropores and increased pore density (Figure 2b). These findings confirm the effective cross‐linking between **HGSM** and **GelMA**, demonstrating that **HGSM** concentration significantly influences the resulting cross‐linking architecture (Figure 2c,d).

**Figure 3:**
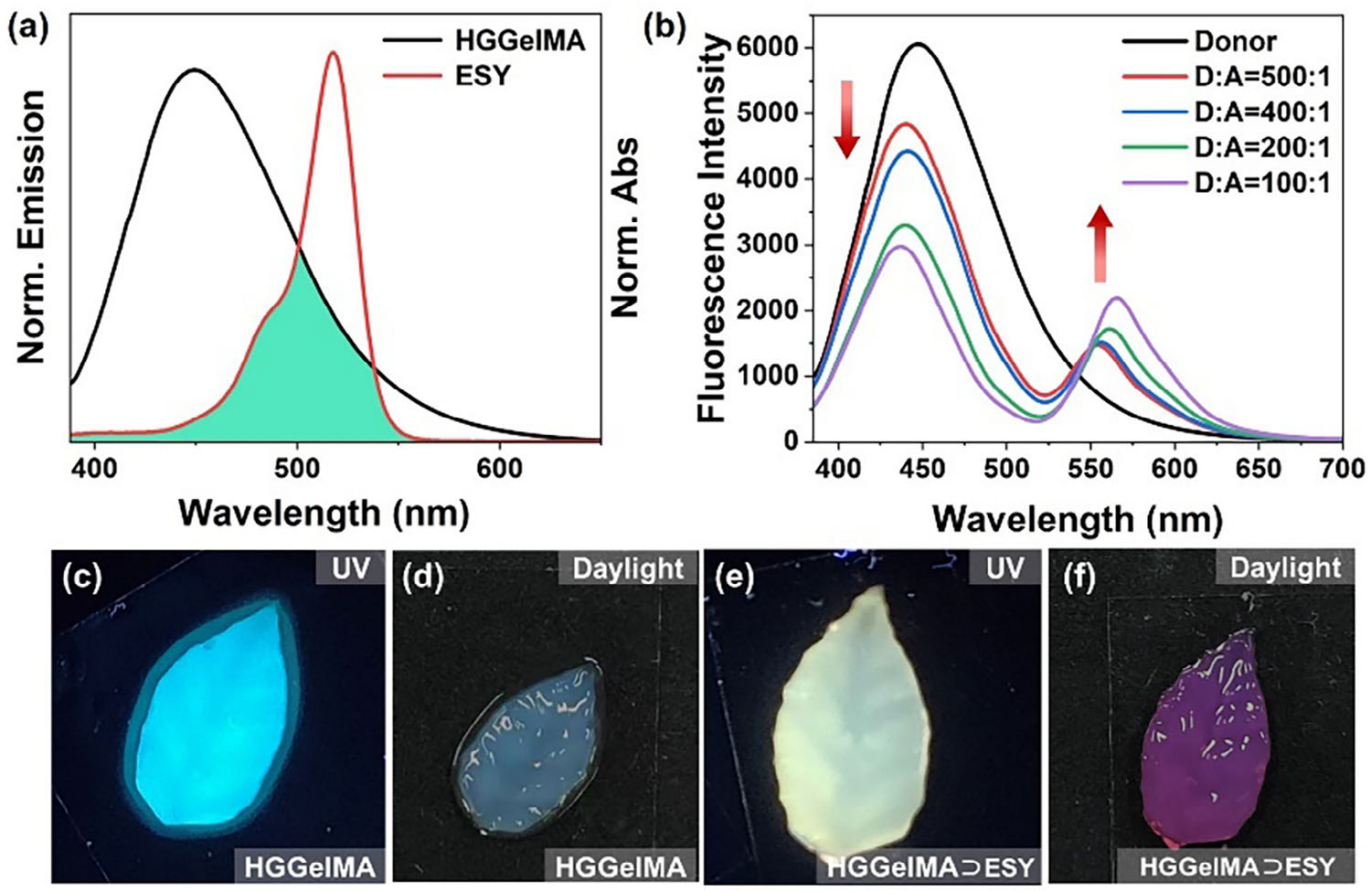
a) Normalized absorption and emission spectra of the **HGGelMA** donor and ESY acceptor. b) Solid‐state fluorescence spectra: **HGGelMA** (donor) and ESY (acceptor). c) and e) Artificial leaf images of **HGGelMA** and **HGGelMA**⊃ESY (100:1) under 365 nm UV light irradiation, respectively. d,f) Artificial leaf images of **HGGelMA** and **HGGelMA**⊃ESY (100:1) under daylight illumination, respectively.

The photophysical characterization of **HGGelMA** revealed a measurable fluorescence quantum yield (Figure S10, Supporting Information). Combined with its aqueous solubility and structural stability, this property enables **HGGelMA** a suitable energy donor for LHS applications in aqueous environments. ESY, a common fluorescent dye, exhibits extensive overlap between its UV absorption spectrum and the emission spectrum of ***m*‐TPEWP5**⊃**G**, making it an ideal energy acceptor (**Figure** **3**a). Moreover, ESY can be encapsulated within the supramolecular hydrogel matrix through electrostatic interactions, which effectively reduces the distance between **HGGelMA** and ESY, thereby enhancing the efficiency of FRET process. Upon gradual addition of ESY to the prepolymerization solution of **HGGelMA**, a photopolymerization reaction was triggered under UV lamp irradiation, resulting in the formation of **HGGelMA**⊃ESY hydrogels with varying donor‐acceptor ratios. As shown in the solid‐state fluorescence spectra (Figure 3b), increasing concentration of ESY led to a decrease in the fluorescence emission intensity of **HGGelMA** centered at 445 nm, while the fluorescence intensity of ESY at 550 nm increased correspondingly. This shift resulted in a gradual change in fluorescence color from blue‐green to yellow. To qualitatively evaluate the light‐harvesting ability of **HGGelMA**⊃ESY, the energy transfer efficiency was assessed through the fluorescence quenching rate of **HGGelMA** at 445 nm emission. The results showed that the energy transfer efficiency reached 50.8% (Figure S11, Supporting Information), with an antenna effect of 7.8 at a donor/acceptor molar ratio of 100:1 (Figure S12, Supporting Information). Consequently, a biomimetic artificial leaf **HGGelMA**⊃ESY (100:1) was successfully prepared by photoinitiated polymerization (Figure 3e,f).

To maximize the utilization of the energy captured by **HGGelMA**⊃ESY, it was further utilized as an efficient photoreactor to catalyze the dehalogenation of bromoacetophenone derivatives in aqueous solution. Specifically, the photocatalytic dehalogenation of 2‐bromo‐1‐phenylacetophenone (**1**) was performed using a 365 nm UV light source (32 W).^[^ ^19^ ^]^ After 2 h of UV irradiation in a pure aqueous solution with 0.5 mol% of **HGGelMA**⊃ESY, the resultant product, acetophenone (**1a**), was obtained in excellent yield (**Table** **1**), which was quantified from the resultant ^1^H NMR spectra (Figure S16, Supporting Information) using the relative integration method.^[^ ^20^ ^]^

**Table 1:** Conditions of the photocatalysis dehalogenation reaction of bromoacetophenone.

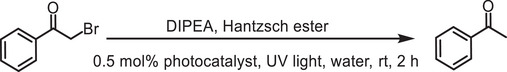			
Entry	Photocatalyst^a)^	Light irradiation	Yield^b)^
1	None	Yes	22%
2	ESY	Yes	54%
3	**HGGelMA**	Yes	28%
4	**HGGelMA**⊃ESY	Yes	>99%
5	**HGGelMA**⊃ESY	No	5%

^a)^ Reaction conditions: bromoacetophenone (20 mg, 0.1 mmol), Hantzsch ester (28 mg, 0.1 mmol), *N*,*N*‐diisopropylethylamine (DIPEA, 35 µL, 0.2 mmol), photocatalyst (0.5% mmol) in water, 32 W UV light, rt, N_2_, 2 h;

^b)^ Product yield was obtained from ^1^H NMR spectra.

To elucidate the specific roles of donors and acceptors in catalytic reactions, we analyzed the outcomes of the reactions conducted under varying conditions (Table 1). The results indicated that in the absence of **HGGelMA** (Figure S14, Supporting Information) or ESY (Figure S15, Supporting Information), the reaction yields were significantly lower even under light irradiation conditions. However, the introduction of **HGGelMA**⊃ESY into the system led to a substantial increase in reaction yield, reaching up to 99%. These experiments collectively validate the high efficiency of **HGGelMA**⊃ESY as a photocatalyst.

We further assess the broad applicability of **HGGelMA**⊃ESY. A series of bromoacetophenone derivatives, encompassing both electron‐donating (compounds **2** and **3**) and electron‐withdrawing substituents (compound **4**), were selected as substrates for the dehalogenation reaction. The experimental results demonstrated that the desired products were obtained in nearly quantitative yields regardless of the substrate employed (**Table** **2**; Figures S18–S20, Supporting Information). Notably, since **HGGelMA**⊃ESY is a solid material, it can be readily recovered from the reaction mixture through a straightforward washing procedure. Moreover, its catalytic efficiency remains robust, highlighting its potential recyclability (Figure S21, Supporting Information).

**Table 2:** The catalytic effects of **HGGelMA**⊃ESY catalysis on various bromoacetophenone derivatives.

Entry	Substrate^a)^	Product	Yield^b)^
1	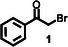	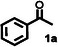	>99%
2	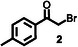	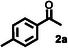	>99%
3	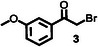	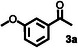	>99%
4	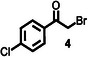	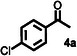	>99%

^a)^ Reaction conditions: substrate (0.1 mmol), Hantzsch ester (28 mg, 0.1 mmol), *N*,*N*‐diisopropylethylamine (DIPEA, 35 µL, 0.2 mmol), **HGGelMA**⊃ESY (0.5% mmol) in water, 32 W UV light, rt, N_2_, 2 h;

^b)^ Product yield was obtained from ^1^H NMR spectra.

Subsequently, to explore the versatility of the biomimetic artificial leaf, we conducted experiments using it in the oxidative coupling reaction of benzylamine. Amines and their derivatives play a critical role as intermediates in drug and fine chemical synthesis. Therefore, we endeavored to leverage the **HGGelMA**⊃ESY system as a heterogeneous catalyst for synthesizing nitrogen‐containing compounds. Since reactive oxygen species (ROS) play an essential role in this process, we initially employed 9,10‐anthracenediyl‐bis(methylene)dimalonic acid (ABDA) and nitro blue tetrazolium (NBT) as specific probes to evaluate the ROS‐generating capacity of the artificial leaf.^[^ ^21^ ^]^ As illustrated in Figures S23 and S24 (Supporting Information), the **HGGelMA**⊃ESY system effectively functions as a type‐II photosensitizer, facilitating the production of ^1^O_2_.

Subsequently, the photocatalytic reaction of benzylamine (**5**) was carried out using a UV lamp as the light source and 0.03 mol% of **HGGelMA**⊃ESY as the catalyst. After 12 h of irradiation in an oxygen‐rich atmosphere, *N*‐benzylidene phenethylamine (**5a**) was selectively obtained with a yield of up to 69% (**Table** **3**), as determined by the internal standard method based on the ^1^H NMR spectra (Figure S28, Supporting Information). Similarly, a series of photocatalytic reactions were performed under varying conditions to elucidate the specific contributions of the donor and acceptor components in the catalytic process (Table 3). In the absence of **HGGelMA** (Figure S26, Supporting Information) or ESY (Figure S27, Supporting Information), the reaction yields were very low even under light irradiation conditions. In contrast, the incorporation of just 0.03 mol% of **HGGelMA**⊃ESY resulted in a remarkable improvement in reaction efficiency.

**Table 3:** Conditions for the photocatalytic oxidative coupling reaction of benzylamine.

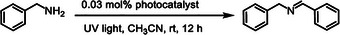			
Entry	Photocatalyst^a)^	Light irradiation	Yield^b)^
1	None	Yes	<1%
2	ESY	Yes	7%
3	**HGGelMA**	Yes	10%
4	**HGGelMA**⊃ESY	Yes	69%
5	**HGGelMA**⊃ESY	No	No reaction

^a)^ Reaction conditions: benzylamine (0.02 mL, 0.18 mmol), acetonitrile (1 mL), photocatalyst (0.03% mmol), 32 W UV light, rt, O_2_, 12 h;

^b)^ Product yield was obtained from ^1^H NMR spectra.

Furthermore, a series of benzylamine derivatives bearing diverse substituents were employed as substrates for the oxidative coupling reaction to verify the generalizability of **HGGelMA**⊃ESY as a catalyst. These derivatives included those modified with either electron‐donating (substrates **6** and **7**) or electron‐withdrawing (substrate **8**) substituents. All of these modifications led to the formation of the anticipated products in relatively high yields, as detailed in **Table** **4** and Figures S30–S32 (Supporting Information). Consistent cycling experiments demonstrated that **HGGelMA**⊃ESY retained its catalytic efficiency after two cycles (Figure S33, Supporting Information).

**Table 4:** The catalytic effects of **HGGelMA**⊃ESY catalysis on various benzylamine derivatives.

Entry	Substrate^a)^	Product^b)^	Yield
1	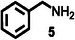	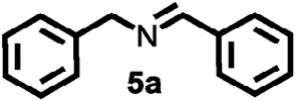	69%
2	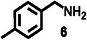	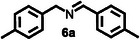	46%
3	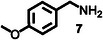	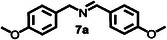	61%
4	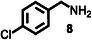	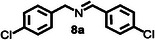	36%

^a)^ Reaction conditions: substrate (0.18 mmol), acetonitrile (1 mL), **HGGelMA**⊃ESY (0.03% mmol), 32 W UV light, rt, O_2_, 12 h;

^b)^ Product yield was obtained from ^1^H NMR spectra.

**Figure 4:**
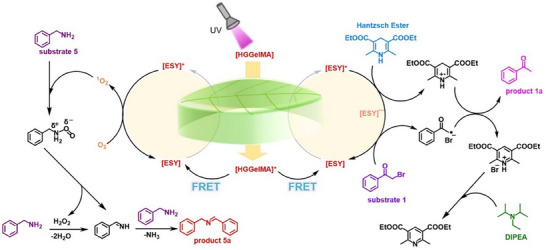
Proposed mechanism of the catalytic dehalogenation and oxidative coupling reaction by using biomimetic artificial leaf systems.

To provide a clearer understanding of the photocatalytic dehalogenation and oxidative coupling reactions, plausible reaction mechanisms were proposed for each process (**Figure** **4**). For the dehalogenation reaction: Initially, the donor **HGGelMA** is irradiated by a 365 nm UV lamp, absorbing photons and transitioning to its excited state **HGGelMA** ^*^. The excited **HGGelMA** ^*^ subsequently transfers energy to ESY through FRET process, promoting ESY to enter its excited state [ESY]^*^. This excited state [ESY]^*^ is then reduced by Hantzsch ester to form the radical anion ESY^•−^. Electrons are subsequently transferred from ESY^•−^ to the substrate 𝛼‐bromoacetophenone, generating the corresponding acetophenone radical, whilst ESY^•−^ is oxidized back to ESY. Finally, the acetophenone radical combines with a hydrogen atom abstracted from the radical cation of the Hantzsch ester, yielding acetophenone as the desired product. The Hantzsch ester undergoes deprotonation in the presence of DIPEA, ultimately forming diethyl 2,6‐lutidine‐3,5‐dicarboxylate.^[^ ^10^, ^20^ ^]^

For the benzylamine oxidative coupling reaction: Following a similar photo‐excitation process, ESY^*^ transfers energy to O_2_, generating ^1^O_2_ and returning ESY to its ground state. Subsequently, ^1^O_2_ captures two protons from benzylamine, converting into H_2_O_2_, while benzylamine is oxidized to form an imine intermediate. This imine then undergoes a nucleophilic addition with another molecule of benzylamine, resulting in the formation of *N*‐benzylidene phenethylamine as the desired product and releasing ammonia (NH_3_) as a byproduct.^[^ ^22^ ^]^

## 3 Conclusion

In summary, a biomimetic artificial leaf based on a supramolecular hydrogel was successfully prepared. The stable hydrogel **HGGelMA** was constructed through non‐covalent interactions between host ***m*‐TPEWP5** and an amphiphilic guest **G**, as well as covalent crosslinking with **GelMA**. Leveraging the FRET process, this artificial leaf serves as an efficient photoreactor to catalyze the dehalogenation of brominated acetophenone derivatives in aqueous solution, achieving a remarkable yield of 99%. Additionally, it can also function as a type‐II photosensitizer, converting oxygen into ^1^O_2_ via photosensitization, which subsequently catalyzes the oxidative coupling of benzylamine derivatives with high efficiency. Notably, this artificial leaf demonstrates outstanding recyclability throughout the catalytic cycle. The present work not only provides a novel approach for the construction of highly efficient AIE‐active light‐harvesting systems but also presents an innovative design strategy for recyclable and soft materials based on *meso*‐functionalized pillararene derivatives for photocatalytic application.

## Experimental Section

### Procedure of Photocatalytic Reaction—Dehalogenation Reaction

Under nitrogen atmosphere, bromoacetophenone (20 mg, 0.1 mmol), Hantzsch ester (28 mg, 0.1 mmol), *N*,*N*‐diisopropylethylamine (DIPEA, 35 µL, 0.2 mmol), **HGGelMA**⊃ESY (0.5% mmol), and deionized water (1 mL) were sequentially added to a quartz flask. Then, the mixture was stirred for 2 h at room temperature under 32 W (365 nm) ultraviolet irradiation. After the reaction, the mixture was extracted with CDCl_3_, and the organic phase was dried over Na_2_SO_4_. The product yield was quantified using ^1^H NMR spectroscopy

### Procedure of Photocatalytic Reaction—Oxidative Coupling Reactions

Under an oxygen atmosphere, benzylamine (0.02 mL, 0.18 mmol), **HGGelMA**⊃ESY (0.03% mmol), and acetonitrile (1 mL) were added to a quartz flask. Then, the mixture was stirred for 12 h at room temperature under 32 W (365 nm) ultraviolet irradiation. After the reaction, 1,3,5‐trimethylbenzene (0.01 mL, 0.07 mmol) was added as an internal standard. The mixture was then extracted with CD_2_Cl_2_ and the organic phase was dried over Na_2_SO_4_. The yield was subsequently determined by quantitative ^1^H NMR analysis.

### Procedures for Recycling Process

Upon completion of the photocatalytic reaction, the organic phase was extracted and subsequently dried over Na_2_SO_4_. Thereafter, **HGGelMA**⊃ESY was isolated from the aqueous phase via filtration and further subjected to three consecutive rinses with deionized water. Finally, **HGGelMA**⊃ESY was dried for reuse in subsequent reactions.

### Statistical Analysis

Figure 3a shows the normalized **HGGelMA** donor emission spectrum and the absorption spectrum of ESY acceptor.

## Conflict of Interest

The authors declare no conflict of interest.

## Supporting information



Supporting Information

## Data Availability

The data that support the findings of this study are available in the supplementary material of this article.
