# Supplementary material for: Bionic Artificial Leaves Based on AIE‐Active Supramolecular Hydrogel for Efficient Photocatalysis
Source: Adv Sci (Weinh). 2025 May 8;12(28):2504993. doi: 10.1002/advs.202504993 (PMC12302627; doi:10.1002/advs.202504993)
Supplement: Supplementary file 1 — Supporting Information [file ADVS-12-2504993-s001.docx]

Supporting Information
©Wiley-VCH 2021
69451 Weinheim, Germany

Bionic Artificial Leaves Based on AIE-active Supramolecular Hydrogel for Efficient Photocatalysis

Rongbo Zhang,^†^ Xueqi Tian,^†^ Minzan Zuo,^†^ Tao Zhang, Srikala Pangannaya, Xiao-Yu Hu*

**Abstract:** A novel hydrogel-based biomimetic artificial leaf is fabricated by integrating host-guest interactions with covalent bonding. Specifically, a water-soluble tetraphenylethylene-embedded pillar[5]arene (***m*-TPEWP5**), which exhibits aggregation-induced emission property, is synthesized as the host molecule. An amphiphilic guest **G** is introduced to form a stable complex (**HGSM**) via non-covalent interactions. Subsequent copolymerization of **HGSM** with gelatin methacryloyl (**GelMA**) yields a hydrogel network (**HGGelMA**), which not only exhibits AIE characteristics but also enables encapsulation of the acceptor eosin Y (ESY), thereby resulting in the construction of an artificial light-harvesting system **HGGelMA**⊃ESY that serves as a biomimetic leaf. To emulate natural photosynthesis more closely and optimize the utilization of the collected energy, two organic reactions are performed within this artificial leaf: dehalogenation of bromoacetophenone derivatives and coupling of benzylamine. These reactions demonstrate remarkable catalytic activity and recycling ability during the photocatalytic process.

Table of Contents

[1. General information 3](#_Toc192788513)

[2. Synthesis of host molecule ***m*-TPEWP5** and gust molecule **G** 3](#_Toc192788514)

[3. Self-assembly of ***m*-TPEWP5** with **G_M_** 8](#_Toc192788515)

[4. Preparation of **GelMA**, **HGSM**, and **HGGelMA** 9](#_Toc192788516)

[5. Fluorescence lifetimes of supramolecular hydrogel 10](#_Toc192788517)

[6. Fluorescence quantum yields of supramolecular hydrogel 10](#_Toc192788518)

[7. Energy transfer efficiency calculation 11](#_Toc192788519)

[8. Antenna effect (AE) calculation 11](#_Toc192788520)

[9. Investigation of dehalogenation reaction in aqueous solution 12](#_Toc192788521)

[10. Proposed mechanism for the 2-bromo-1-phenylethanone dehalogenation reaction 16](#_Toc192788522)

[11. Investigation of ^1^O_2_ generation 17](#_Toc192788523)

[12. Investigation of oxidative coupling reactions 18](#_Toc192788524)

[10. Proposed reaction mechanism for the oxidative coupling of benzylamines 22](#_Toc192788525)

[11. References 23](#_Toc192788526)

[Author Contributions 23](#_Toc192788527)

1. General information

The commercially available reagents and solvents were either employed as purchased or dried according to procedures described in the literatures. All reactions were performed under nitrogen atmosphere unless otherwise stated. Analytical thin layer chromatography (TLC) was performed using 0.25 mm silica gel plates. Column chromatography was performed with silica gel (200-300 mesh) produced by Shanghai Titan Scientific Co., Ltd. All yields were given as isolated yields. ^1^H and ^13^C NMR spectra were recorded on a Bruker Avance 400 MHz spectrometer with internal standard tetramethylsilane (TMS) and solvent signals as internal references at 298 K, and the chemical shifts (δ) were reported in ppm and coupling constant (*J*) values were given in Hz. High-resolution electrospray ionization mass spectra (HR-ESI-MS) were recorded on an Orbitrap Exploris 120 equipped with an electrospray ionization (ESI) probe operating in positive-ion mode with direct infusion. UV-visible spectra were recorded with a Shimadzu UV 1780 UV-Vis Spectrophotometer. Fluorescence spectra were recorded on a Gangdong SCI F-380 fluorescence spectrophotometer. Scanning electron microscope (SEM) investigations were carried out using a FEI Quanta FEG 250 instrument. The fluorescence lifetimes were measured employing time-correlated single photon counting on a FLS980 instrument (Edinburg Instruments Ltd., Livingstone, UK) with a pulsed xenon lamp. The quantum yields were carried out on a FLS980 instrument with the integrating sphere.

2. Synthesis of host molecule *m*-TPEWP5 and gust molecule G

**2.1. Synthesis of host molecule *m*-TPEWP5**

***m*-TPTWP5** was synthesized according to previously reported procedures^[1]^.

**Scheme** **S1**. Synthesis route of host molecule ***m*-TPEWP5**.

**2.2. Synthesis of guest molecule G**

**Scheme** **S2**. Synthesis route of guest molecule **G**.

2.2.1 *Synthesis of compound* ***6***^[2]^

Tetraethylene glycol (1.5 g, 7.7 mmol) and triethylamine (0.5 mL, 3.85 mmol) were dissolved in anhydrous CH_2_Cl_2_ (4 mL). After cooling to 0 °C, acryloyl chloride (0.25 mL, 3.2 mmol) in 10 mL of anhydrous CH_2_Cl_2_ was added dropwise into the mixture under nitrogen atmosphere. The reaction mixture was stirred at 0 °C for 1 h, and then at room temperature for 4 h after completing the dropwise addition. After quenching with H_2_O (20 mL), the crude product was washed with water and saturated brine several times. The organic phase was dried over anhydrous Na_2_SO_4_ and the solvent was removed under vacuum. The product was purified by column chromatography using DCM/MeOH (20:1, *v*/*v*) as eluent to give compound **6** as a colorless oil (0.34 g, 1.37 mmol, yield: 43%). ^1^H NMR (400 MHz, CDCl_3_, 298 K) δ 6.42 (d, *J* = 17.3 Hz, 1H), 6.15 (dd, *J* = 17.3, 10.4 Hz, 1H), 5.83 (d, *J* = 10.4 Hz, 1H), 4.35 – 4.29 (m, 2H), 3.77 – 3.70 (m, 4H), 3.66 (s, 8H), 3.63 – 3.56 (m, 2H).

**
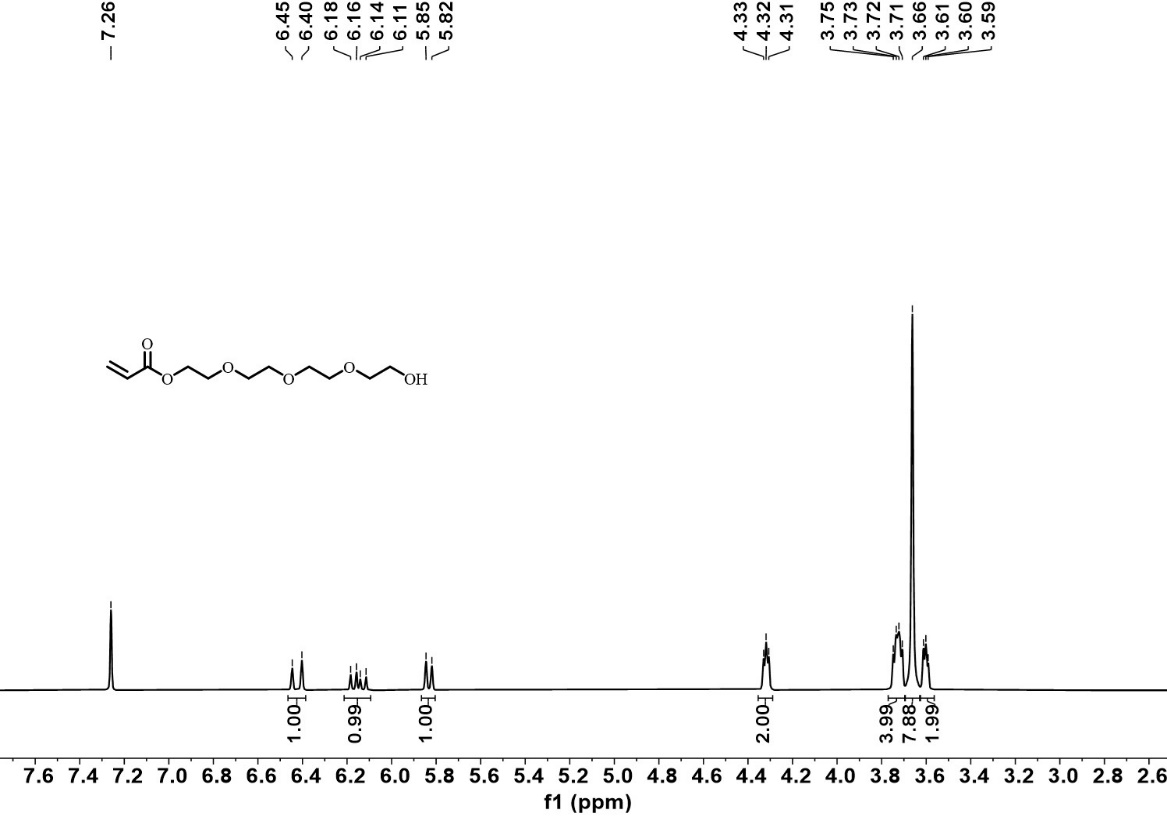
**

**Figure S1.** ^1^H NMR spectrum (400 MHz, CDCl_3_, 298 K) of compound **6**.

2.2.2 *Synthesis of compound* ***7***

A solution of 5-hydroxy-isophthalic acid dimethyl ester (0.50 g, 2.38 mmol) in CH_3_CN (10 mL) was treated with K_2_CO_3_ (0.65 g, 4.76 mmol). The resulting reaction mixture was stirred under reflux for 30 minutes followed by the addition of propargyl bromide (0.22 mL, 2.85 mmol). After stirring for 24 h under reflux, the reaction mixture was concentrated under vacuum. The residue was taken up in ethyl acetate (50 mL), washed successively with water (20 mL) and brine (20 mL), dried over anhydrous sodium sulphate and concentrated under vacuum. The crude product was then recrystallized from dichloromethane and hexane by slow evaporation (20 mL, 2:1, *v*/*v*) to provide compound **7** as a white crystalline solid (0.56 g, 2.26 mmol, yield: 95%). ^1^H NMR (400 MHz, CDCl_3_, 298 K) δ 8.33 (s, 1H), 7.83 (s, 2H), 4.79 (s, 2H), 3.95 (s, 6H), 2.55 (s, 1H).

**
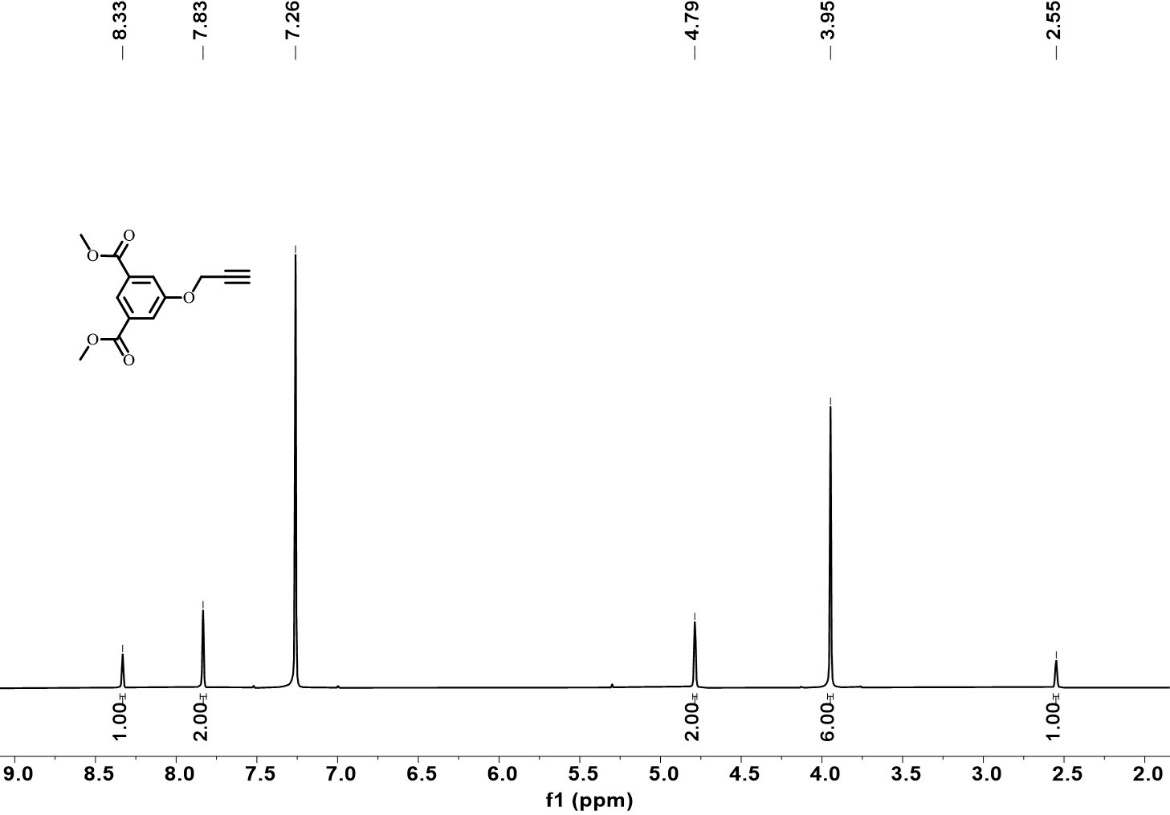
**

**Figure S2.** ^1^H NMR spectrum (400 MHz, CDCl_3_, 298 K) of compound **7**.

2.2.3 *Synthesis of compound* ***8***

A solution of compound **7** (0.3 g, 1.2 mmol) in methanol (5 mL) was treated with KOH (0.3 g, 4.8 mmol). The resulting reaction mixture was stirred overnight under reflux. After quenching with water, the mixture was extracted with dichloromethane (10 mL) and the aqueous phase was acidified to pH< 2 using 6 M HCl. The precipitate was collected by filtration. The solid product was dried in vacuo, and the compound **8** was obtained as a white solid (0.26 g, 1.18 mmol, yield: 90%). ^1^H NMR (400 MHz, DMSO-*d*_6_, 298 K) δ 13.30 (s, 2H), 8.11 (s, 1H), 7.72 (d, *J* = 1.0 Hz, 2H), 4.95 (d, *J* = 2.2 Hz, 2H), 3.64 (t, *J* = 2.1 Hz, 1H).


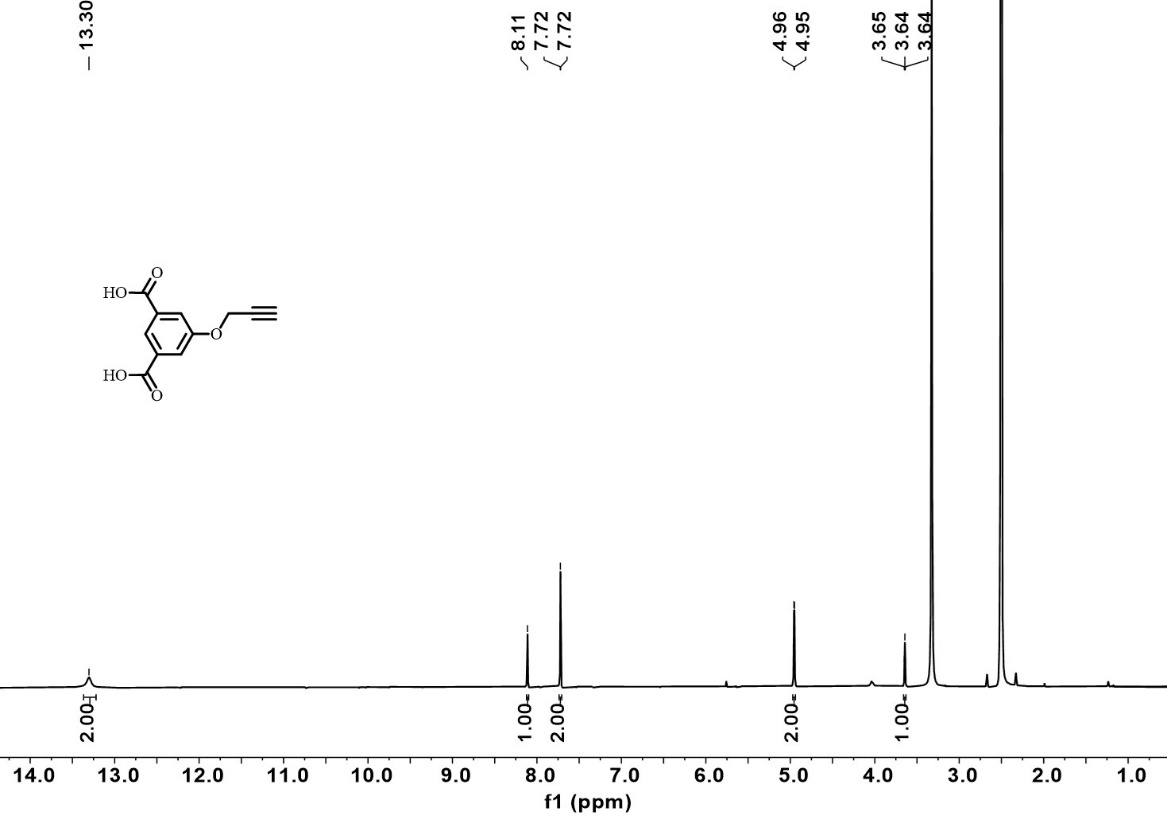


**Figure S3.** ^1^H NMR spectrum (400 MHz, DMSO-*d_6_* 298 K) of compound **8**.

2.2.4 *Synthesis of compound* ***9***

Compound **8** (0.2 g, 0.91 mmol) were dissolved in 4 mL of dichloromethane and 0.2 mL of DMF. After cooling to 0 °C, oxalyl chloride (0.23 g, 1.82 mmol) was added dropwise into the mixture under nitrogen atmosphere. The reaction mixture was stirred at 0 °C for 5 min, and then at 30 °C for 3 h after completing the dropwise addition. After removal of the solvent under reduced pressure get compound **9** as brown solid (0.23 g, 0.89 mmol, yield: 98%).

2.2.5 *Synthesis of compound* ***10***

Compound **6** (0.56 g, 2.25 mmol) and triethylamine (0.15 mL) were dissolved in 2 mL of dichloromethane under nitrogen atmosphere. The reaction solution was cooled to 0 °C using an ice bath. Compound **9** (0.23 g, 0.90 mmol) was then dissolved in 2 mL of dichloromethane and slowly added to the reaction mixture. After the addition, the reaction mixture was stirred at room temperature for 12 h, following completion of the reaction, the mixture was filtered and then concentrated under vacuum. The product was purified by column chromatography using DCM/MeOH (15:1, *v*/*v*) to get compound **10** as a yellow oil (0.35 g, 0.52 mmol, yield: 58%). ^1^H NMR (400 MHz, CDCl_3_, 298 K) δ 8.30 (s, 1H), 7.80 (s, 2H), 6.38 (d, *J* = 17.4 Hz, 2H), 6.10 (dd, *J* = 17.2, 10.4 Hz, 2H), 5.79 (d, *J* = 10.4 Hz, 2H), 4.75 (s, 2H), 4.46 (t, *J* = 5.2 Hz, 4H), 4.26 (t, *J* = 5.0 Hz, 4H), 3.80 (t, *J* = 5.2 Hz, 4H), 3.71 – 3.59 (m, 20H), 2.57 (s, 1H).


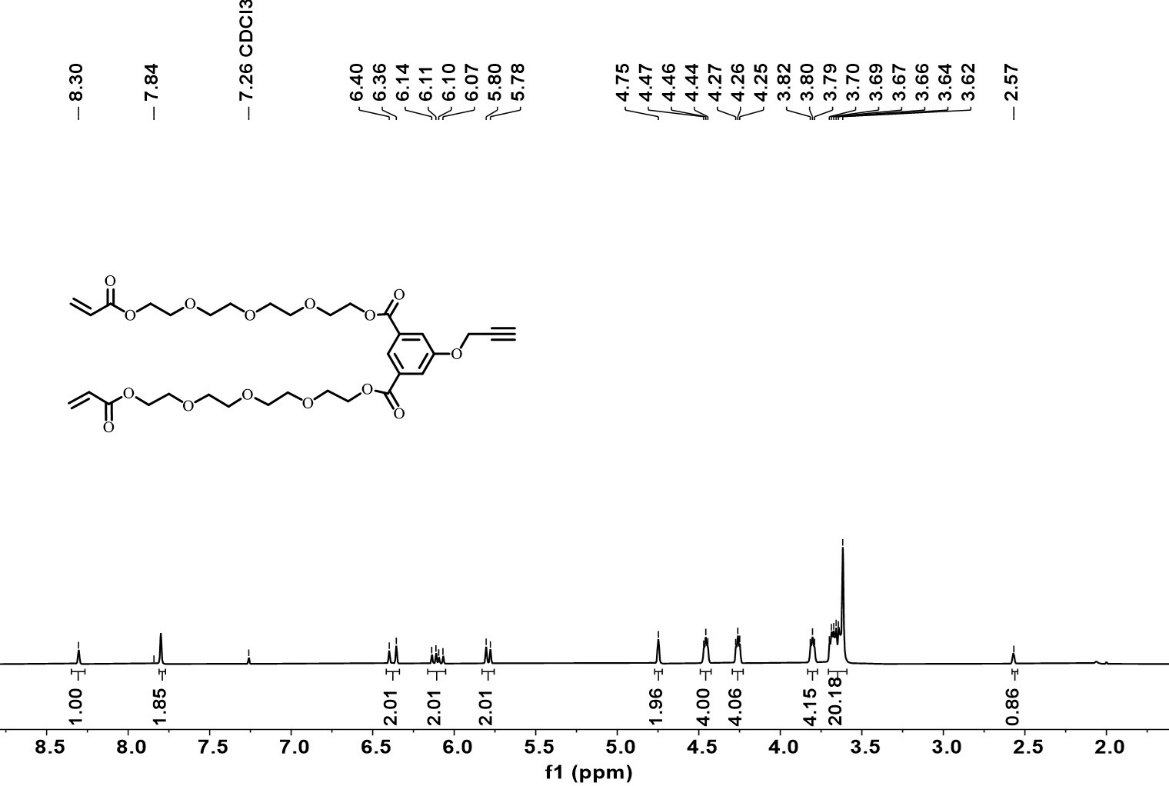


**Figure S4.** ^1^H NMR spectrum (400 MHz, CDCl_3_, 298 K) of compound **10**.

2.2.6 *Synthesis of compound* ***G***

A mixture of N_3_(CH_2_)_6_SO_3_Na (0.05 g, 0.2 mmol), tris-(benzyltriazolylmethyl)amine (0.01 g, 0.017 mmol) and Cu(CNCH_3_)_4_PF_6_ (0.006 g, 0.017 mmol) were dissolved in methanol (3 mL), and a solution of compound **10** (0.12 g, 0.18 mmol) in dichloromethane was added. The reaction mixture was stirred under nitrogen atmosphere for 24 h at room temperature. After removal of the solvent under reduced pressure, the product was purified by column chromatography using DCM/MeOH (20:1, *v*/*v*) to afford compound **G** as a green oil (0.13 g, 0.14 mmol, yield: 80%). ^1^H NMR (400 MHz, D_2_O, 298 K) δ 8.10 (s, 1H), 8.04 (s, 1H), 7.68 (s, 2H), 6.30 (d, *J* = 17.2 Hz, 2H), 6.03 (dd, *J* = 17.2, 10.5 Hz, 2H), 5.86 (d, *J* = 10.4 Hz, 2H), 5.22 (s, 2H), 4.49 – 4.42 (m, 4H), 4.41 – 4.34 (m, 2H), 4.16 – 4.09 (m, 4H), 3.90 – 3.82 (m, 4H), 3.72 – 3.68 (m, 4H), 3.65 – 3.57 (m, 12H), 3.56 – 3.53 (m, 4H), 2.84 – 2.76 (m, 2H), 1.83 (t, *J* = 7.5 Hz, 2H), 1.63 (t, *J* = 7.6 Hz, 2H), 1.34 (t, *J* = 7.5 Hz, 2H), 1.24 – 1.18 (m, 2H). ^13^C NMR (100 MHz, D_2_O, 298 K) δ 168.03, 166.55, 156.85, 132.32, 131.34, 127.12, 122.77, 121.36, 69.89, 69.70, 69.57, 68.42, 68.30, 64.84, 63.76, 61.52, 50.79, 50.36, 29.13, 27.00, 25.04, 23.76. HR-ESI-MS: m/z [M – Na]^-^ calcd for [C_39_H_56_N_3_O_18_S]^-^ 886.3280, found 886.3242.


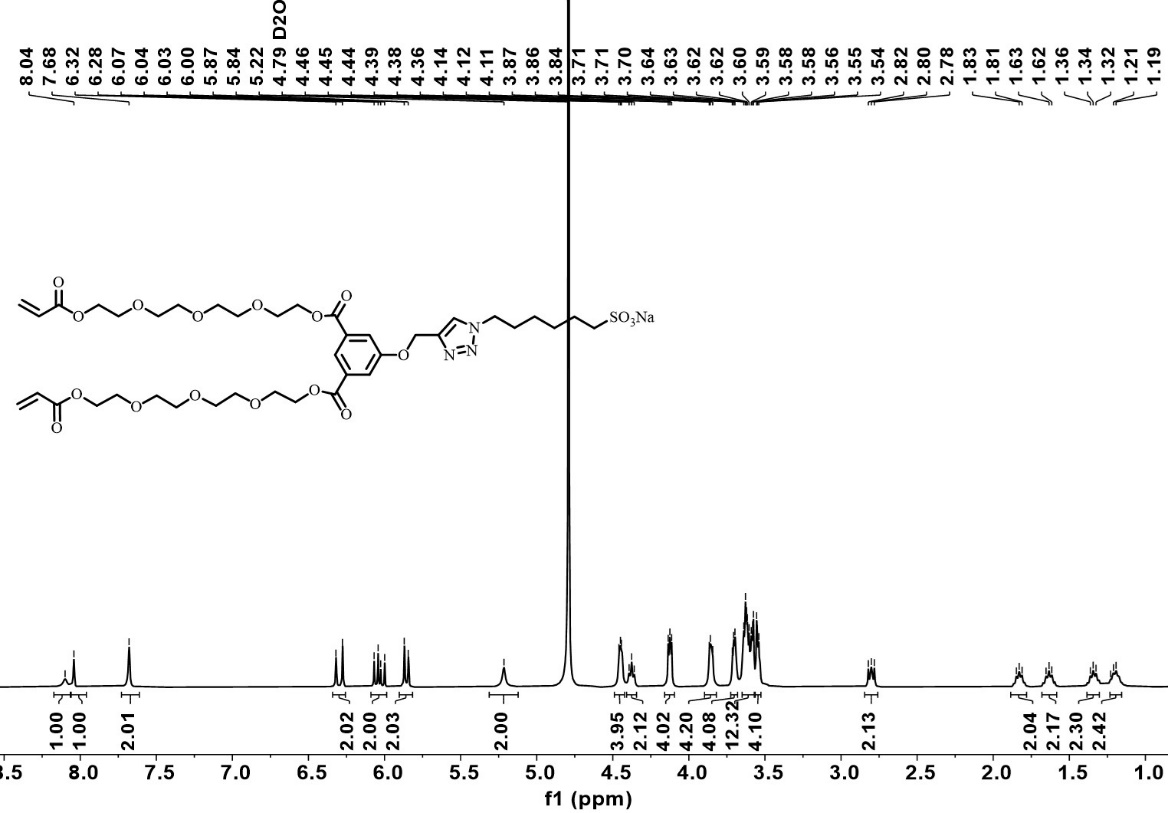


**Figure S5.** ^1^H NMR spectrum (400 MHz, D_2_O, 298 K) of compound **G**.


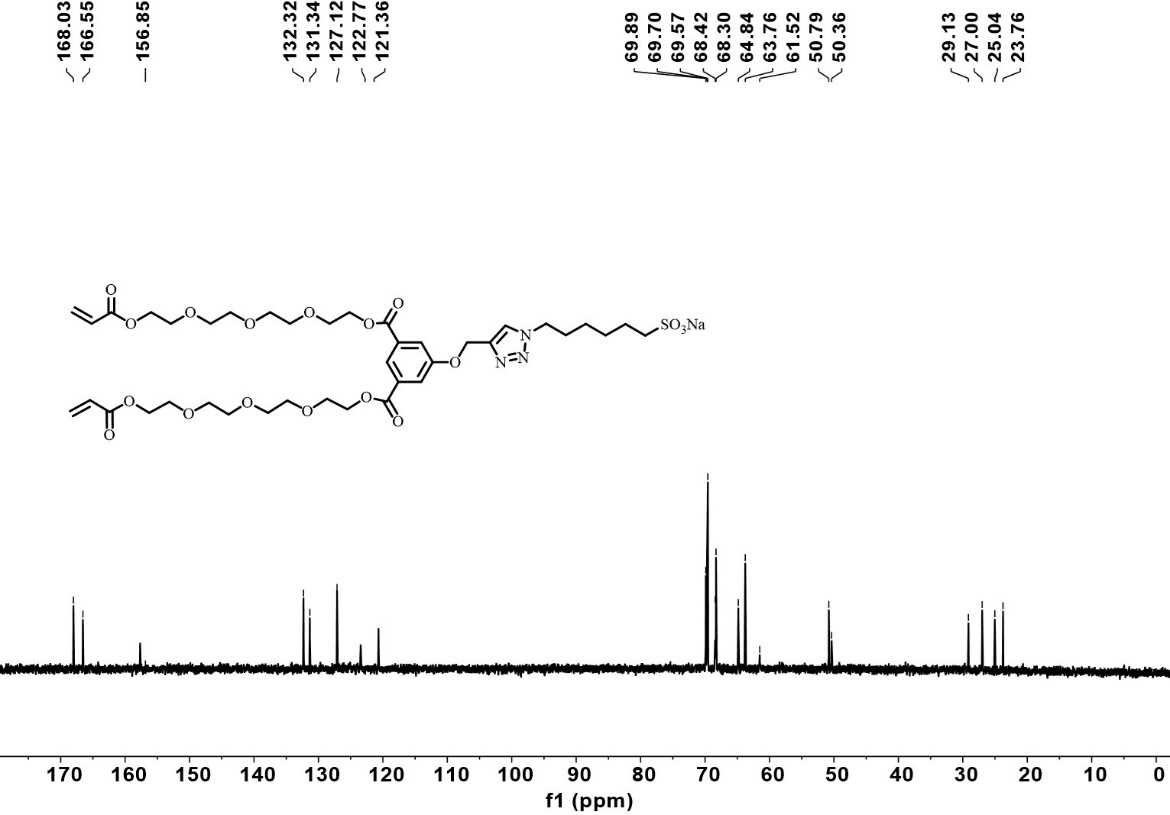


**Figure S6.** ^13^C NMR spectrum (100 MHz, D_2_O, 298 K) of compound **G**.


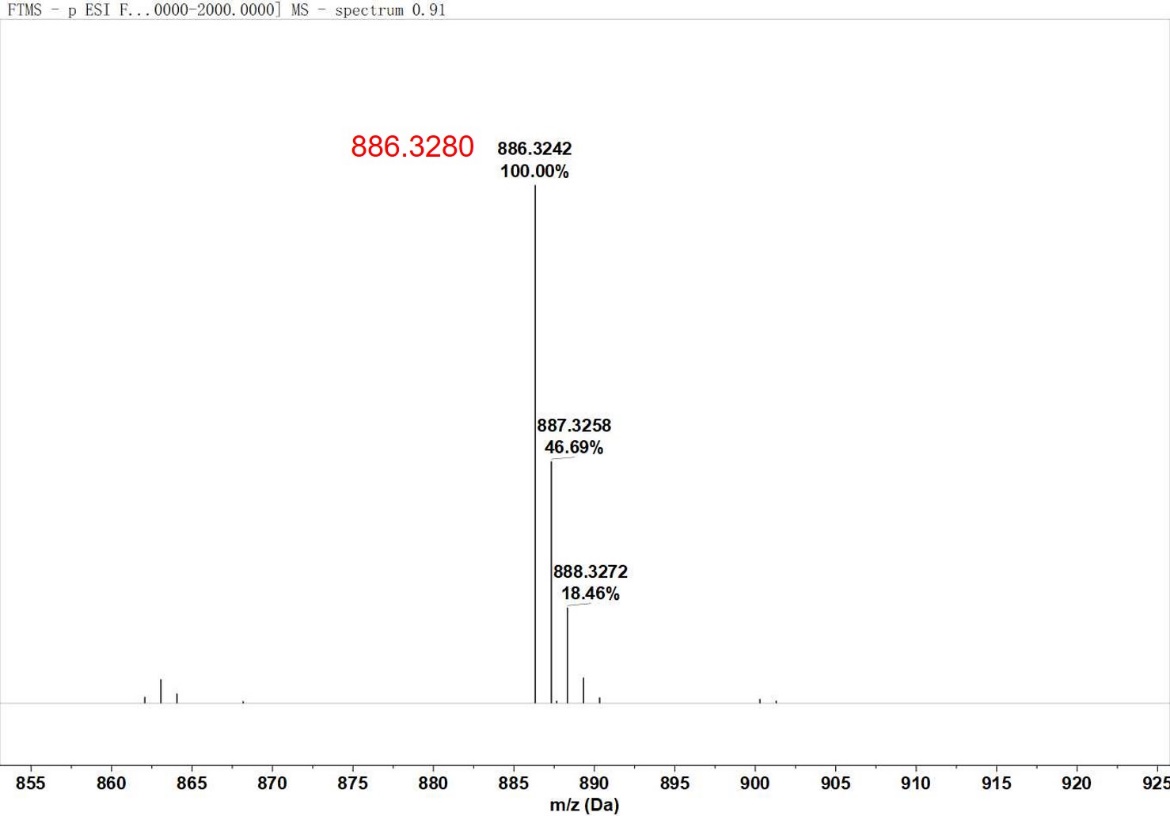


**Figure S7.** HR-ESI-MS spectrum of compound **G**.

3. Self-assembly of *m*-TPEWP5 with G_M_


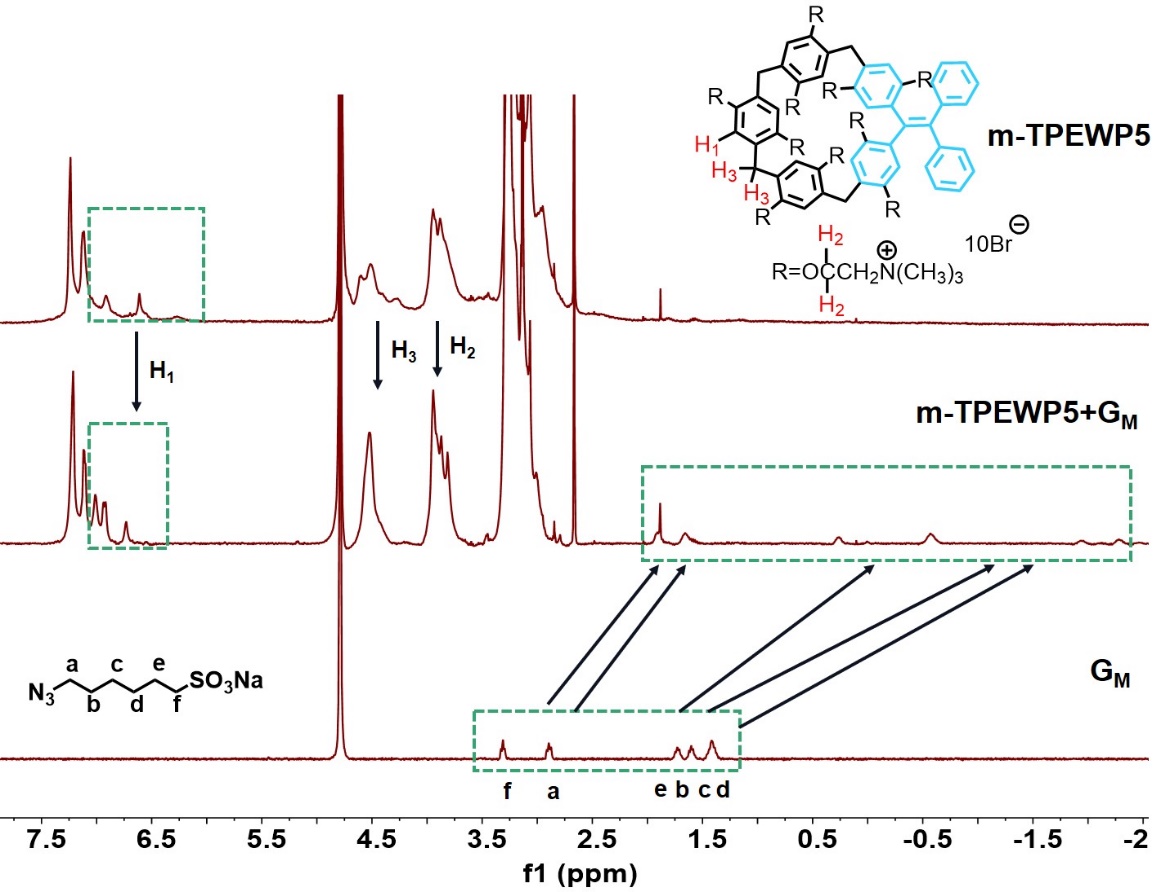


**Figure S8.** ^1^H NMR (400 MHz, D_2_O, 298 K) spectra: ***m*-TPEWP5** (6.0 mM), ***m*-TPEWP5** (6.0 mM) + **G_M_** (6.0 mM), and **G_M_** (6.0 mM).

4. Preparation of GelMA, HGSM, and HGGelMA

**4.1 Synthesis of GelMA**^[3]^

**GelMA** was synthesized by methacrylic anhydride reaction with Gelatin, and it was obtained as a white porous foam after lyophilization (–80 °C, 2 days) and was stored at –20 °C. The ^1^H NMR spectra of **GelMA** in D_2_O were recorded. The actual degree of methacryloyl modification was calculated as follows: Methacryloyl modification (%) = (Number of methacryloyl groups in **GelMA**/Number of amine groups in the unreacted polymers) × 100%


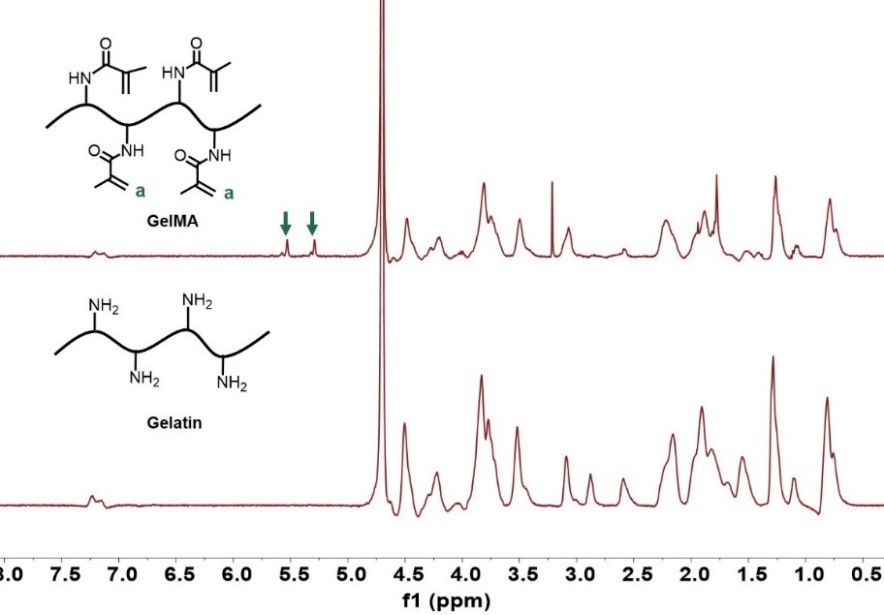


**Figure S9.** ^1^H NMR (400 MHz, D_2_O, 298 K) spectra: **GelMA** (6.0 mM), Gelatin (6.0 mM)

4.2 Preparation of host-guest supramolecules complex HGSM

The ***m*-TPEWP5** (0.27 g, 0.11 mmol) was fully dissolved in 8.8 mL of deionized water, and 0.1 g of oily **G** was added, followed by stirring at room temperature for 24 h. The gradual disappearance of the interface between the oil and water and the formation of a homogeneous and transparent solution indicated that the complexation process between the ***m*-TPEWP5** host and the oily guest **G** was completed. Freeze-drying was performed to obtain a white powder of the host-guest supramolecular complex **HGSM**.

4.3 Preparation of supramolecules hydrogel HGGelMA

**GelMA** was dissolved in deionized water to achieve a concentration of 0.045 mol/L. Subsequently, different concentrations of host-guest complex (0.015 mol/L) and (0.045 mol/L) were added to the **GelMA** solution, respectively for the preparation of hydrogels with different cross-linking densities. Additionally, 0.0015 mol/L of I2959 was included as a photoinitiator. Finally, the mixed solution was injected into a leaf-shaped mold and subsequently exposed to ultraviolet light (365 nm wavelength, 32 W intensity) for cross-linking. After 8 minutes of irradiation, a stable supramolecular hydrogel (**HGGelMA**) was successfully obtained. Hydrogels incorporating different concentrations of host-guest supramolecular crosslinkers were designated as **HG_x_GelMA**, where x represents the concentration of **HGSM** (in mol/L). Unless otherwise specified, **HGGelMA** were prepared using a **HGSM** concentration of 0.015 mol/L.

5. Fluorescence lifetimes of supramolecular hydrogel

**Table S1.** Fluorescence lifetimes of **HGGelMA** and **HGGelMA⸧**ESY **(100:1)** monitored at 445 nm upon excitation at 365 nm.

| Sample | τ_1_ /ns | Rel/% | τ_2_ /ns | Rel/% | τ /ns | χ^2^ |
| --- | --- | --- | --- | --- | --- | --- |
| **HGGelMA** | 1.1192 | 55.62 | 4.5310 | 44.38 | 2.6332 | 1.1325 |
| **HGGelMA**⸧ESY | 1.0947 | 51.52 | 4.2938 | 48.48 | 2.6440 | 1.1310 |

6. Fluorescence quantum yields of supramolecular hydrogel


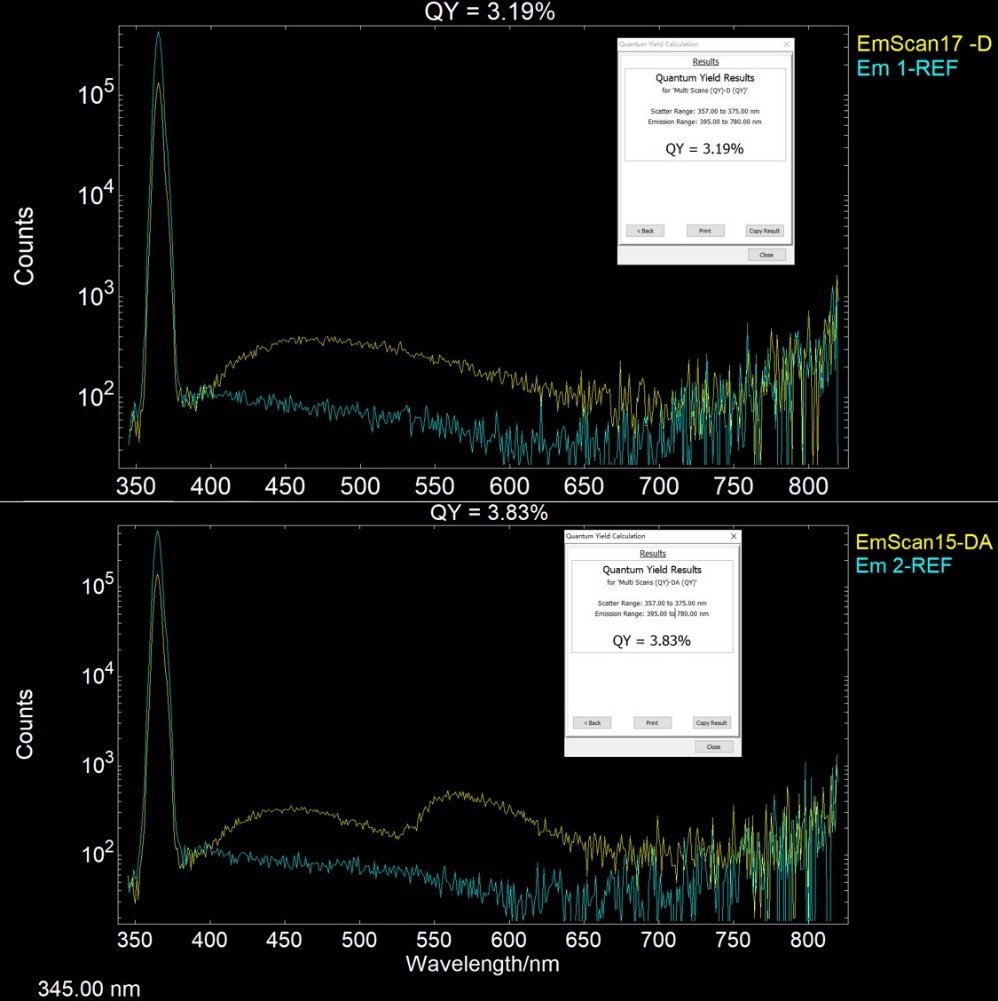


**Figure S10.** Absolute fluorescence quantum yields (Φ_f(abs)_) of (a) **HGGelMA** (b) **HGGelMA**⸧ESY (100:1).

7. Energy transfer efficiency calculation


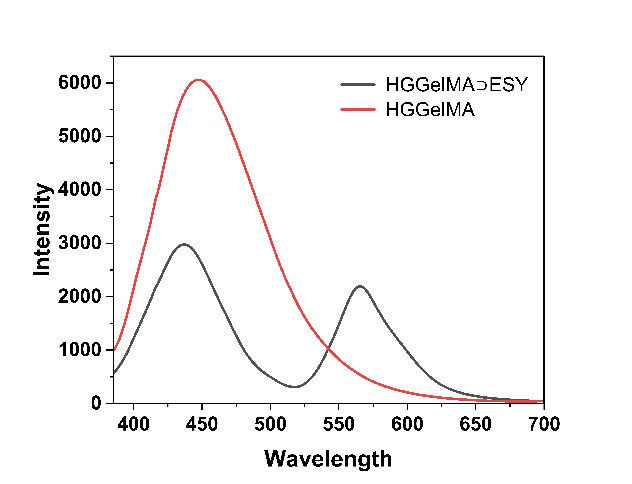


**Figure S11.** Solid-state fluorescence spectra of **HGGelMA** and **HGGelMA**⸧ESY(100:1).

The energy-transfer efficiency (Φ_ET_) was calculated using equation S1:

Φ_ET_ = 1 ‒ I_DA_ / I_D_ (eq. S1)

Where I_DA_ and I_D_ are the fluorescence intensities of the emission of **HGGelMA**⸧ESY (acceptor) and **HGGelMA** (donor), respectively when excited at 365 nm. The energy-transfer efficiency (Φ_ET_) was calculated as 50.8%.^[4, 5]^

8. Antenna effect (AE) calculation


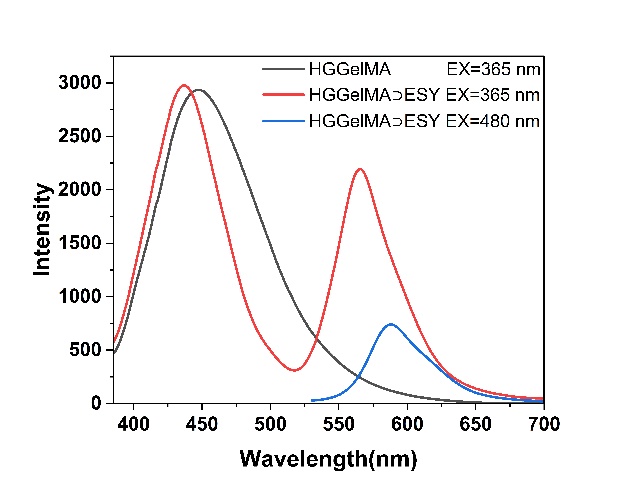


**Figure S12.** Solid-state fluorescence spectra of **HGGelMA**⸧ESY (100:1) in aqueous solution (red line), blue line (acceptor emission, λ_ex_ = 480 nm). The black line represents the fluorescence spectrum of **HGGelMA**, which was normalized according to the fluorescence intensity at 445 nm of the red line.

The antenna effect (AE) was calculated based on the excitation spectra using equation S2:

AE = (I_DA,365_ – I_D,365_) / I_DA,480_ (eq. S2)

Where I_DA,365_ and I_DA,480_ are the fluorescence intensities at 565 nm with the excitation of the donor at 365 nm and the direct excitation of the acceptor at 480 nm, respectively. I_D,365_ is the fluorescence intensity at 565 nm of the **HGGelMA**, which was normalized with the **HGGelMA**⸧ESY assembly at 445 nm. The antenna effect value was calculated as 7.8.^[4, 5]^

9. Investigation of dehalogenation reaction in aqueous solution

Table S2. Dehalogenation reaction of 2-bromo-1-phenylethanone and its derivatives under different reaction conditions.

| Entry | R | Photocatalyst^[a]^ | Light irradiation | Yield^[b]^ |
| --- | --- | --- | --- | --- |
| 1 | H | None | Yes | 22% |
| 2 | H | ESY | Yes | 54% |
| 3 | H | **HGGelMA** | Yes | 28% |
| 4 | H | **HGGelMA**⊃ESY | Yes | >99% |
| 5 | H | **HGGelMA**⊃ESY | No | 5% |
| 6 | *p*-Me | **HGGelMA**⊃ESY | Yes | >99% |
| 7 | *m*-OMe | **HGGelMA**⊃ESY | Yes | >99% |
| 8 | *p*-Cl | **HGGelMA**⊃ESY | Yes | >99% |

[a] Reaction conditions: Bromoacetophenone (20 mg, 0.1 mmol), Hantzsch ester (28 mg, 0.1 mmol), *N*,*N*-diisopropylethylamine (DIPEA, 35 μL, 0.2 mmol), **HGGelMA**⊃ESY (0.5% mmol) in water, 32 W UV light, rt, N_2_, 2 h. [b] Product yield was obtained from ^1^H NMR spectra.

Since **HGGelMA**⊃ESY is a solid gel, CDCl_3_ was selected to extract the product and substrate from the reaction solution after the completion of reaction. In this way, it contains the characteristic peaks of Hantzsch ester and DIPEA in the NMR spectrum. However, for calibration purposes, we specifically selected the -CH_3_ group of the product and the -CH_2_ group of the substrate for calibration. And relative measurements were used to calculate yields: η=(*A_1_*/*n_1_*)/(*A_1_*/*n_1_*+*A_2_*/*n_2_*) %.^[6]^

Herein, *A_1_* represents the integral area of the -CH_3_ group in the product, *n_1_* denotes the number of protons corresponding to this characteristic peak. Similarly, *A_2_* represents the integral area of the -CH_2_ group in the substrate, and *n_2_* denotes the number of protons corresponding to this characteristic peak.


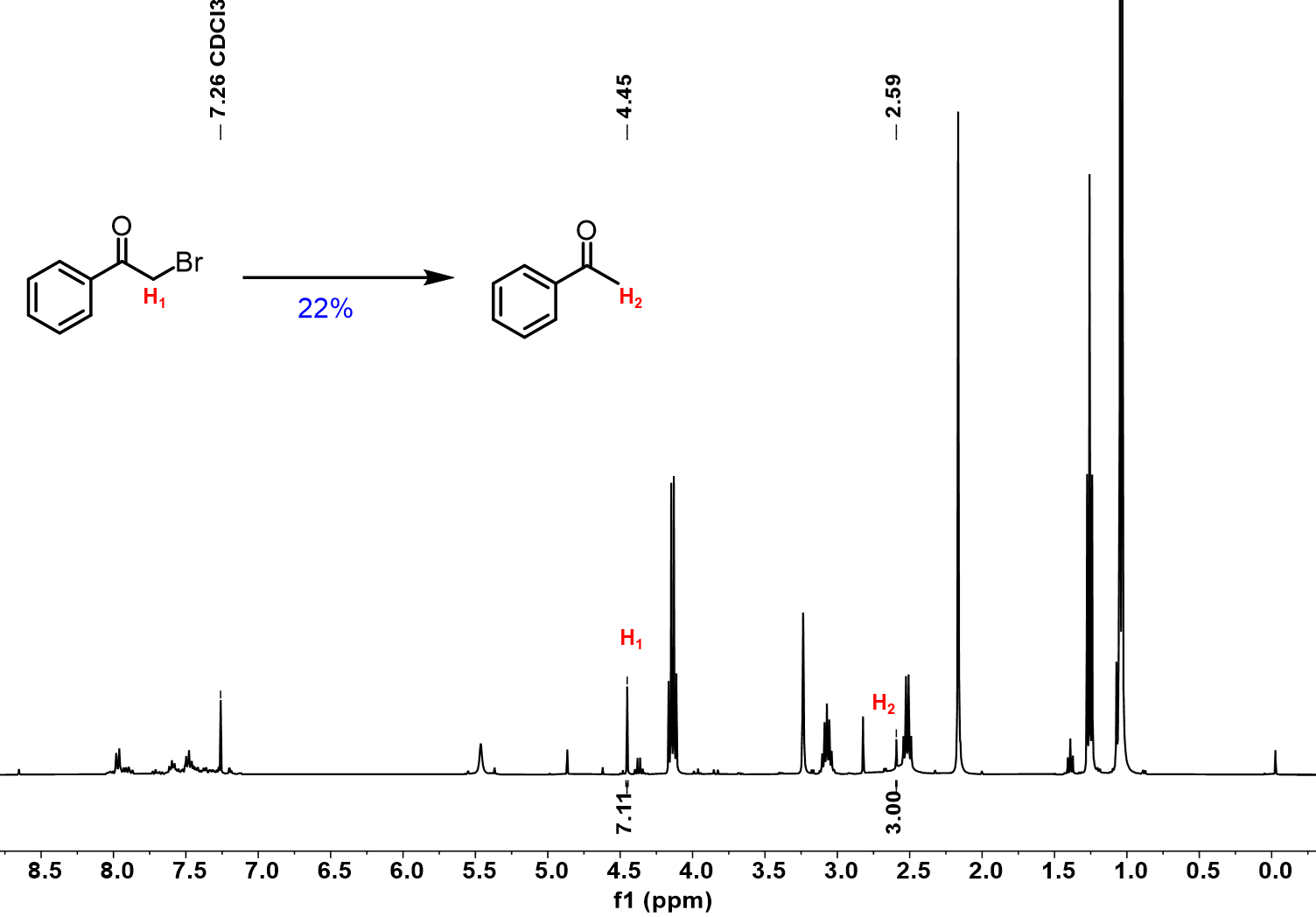


**Figure S13.** ^1^H NMR (400 MHz, CDCl_3_, 298 K) spectrum of the reaction mixture of entry 1.


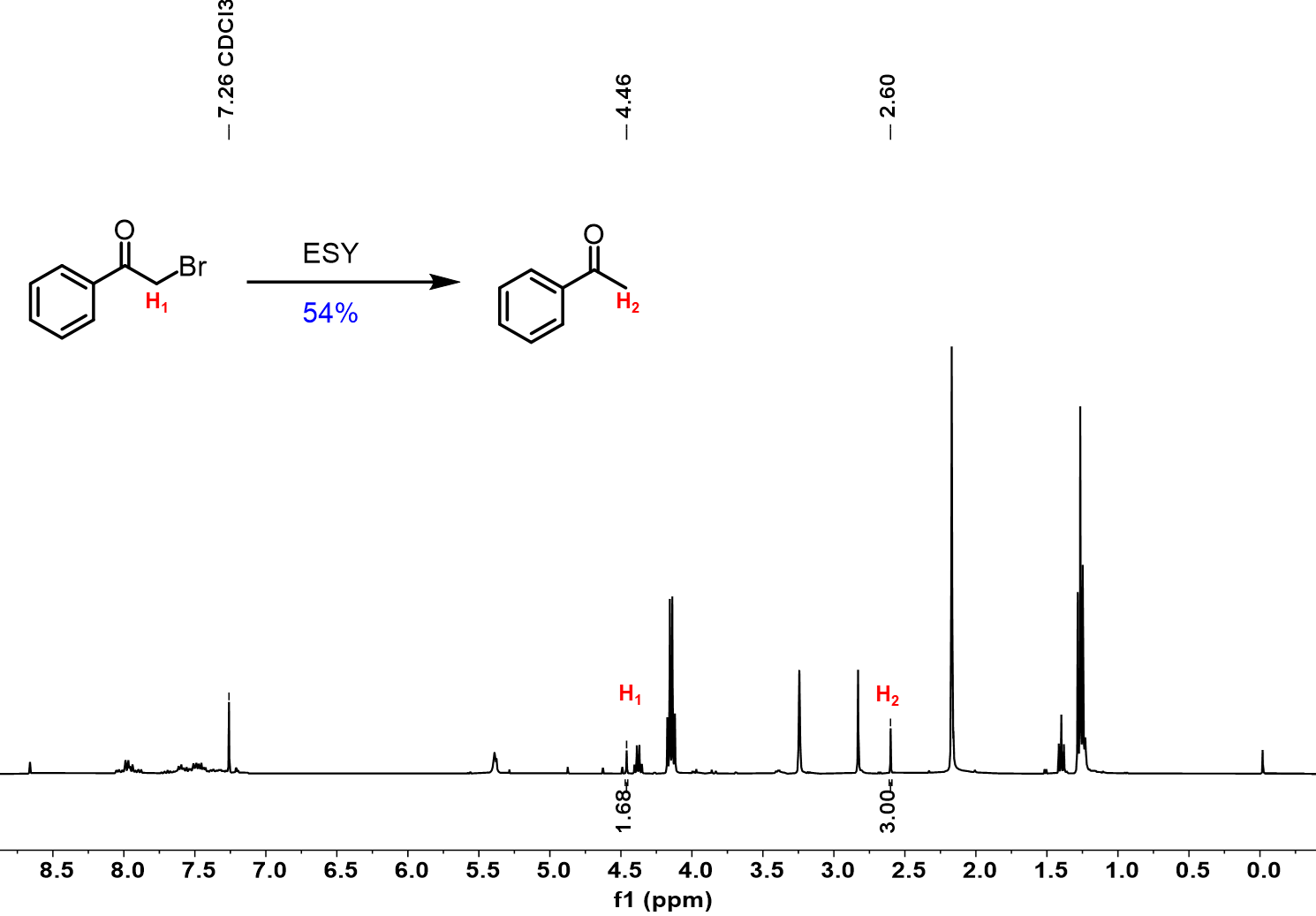


**Figure S14.** ^1^H NMR (400 MHz, CDCl_3_, 298 K) spectrum of the reaction mixture of entry 2.


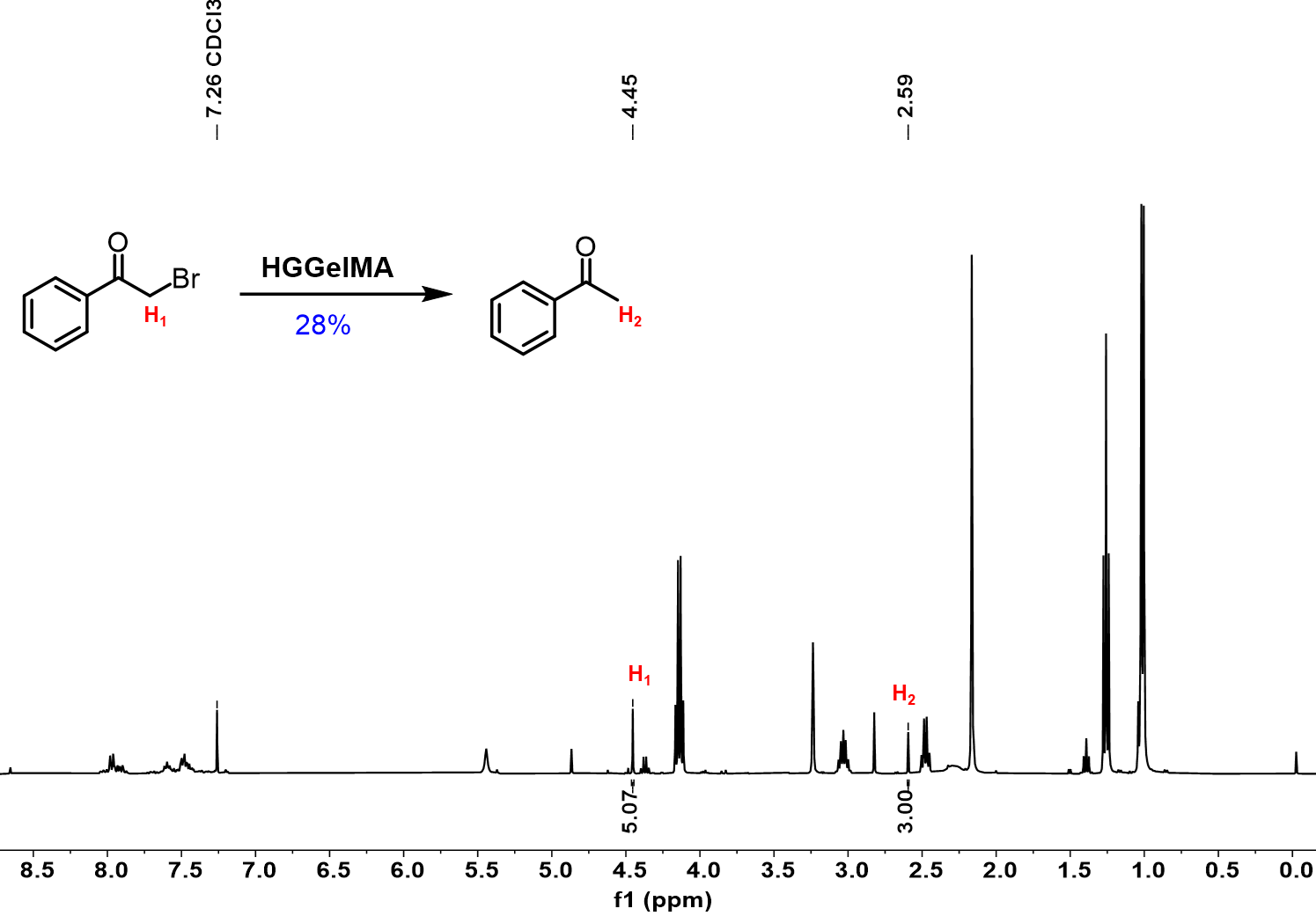


**Figure S15.** ^1^H NMR (400 MHz, CDCl_3_, 298 K) spectrum of the reaction mixture of entry 3.


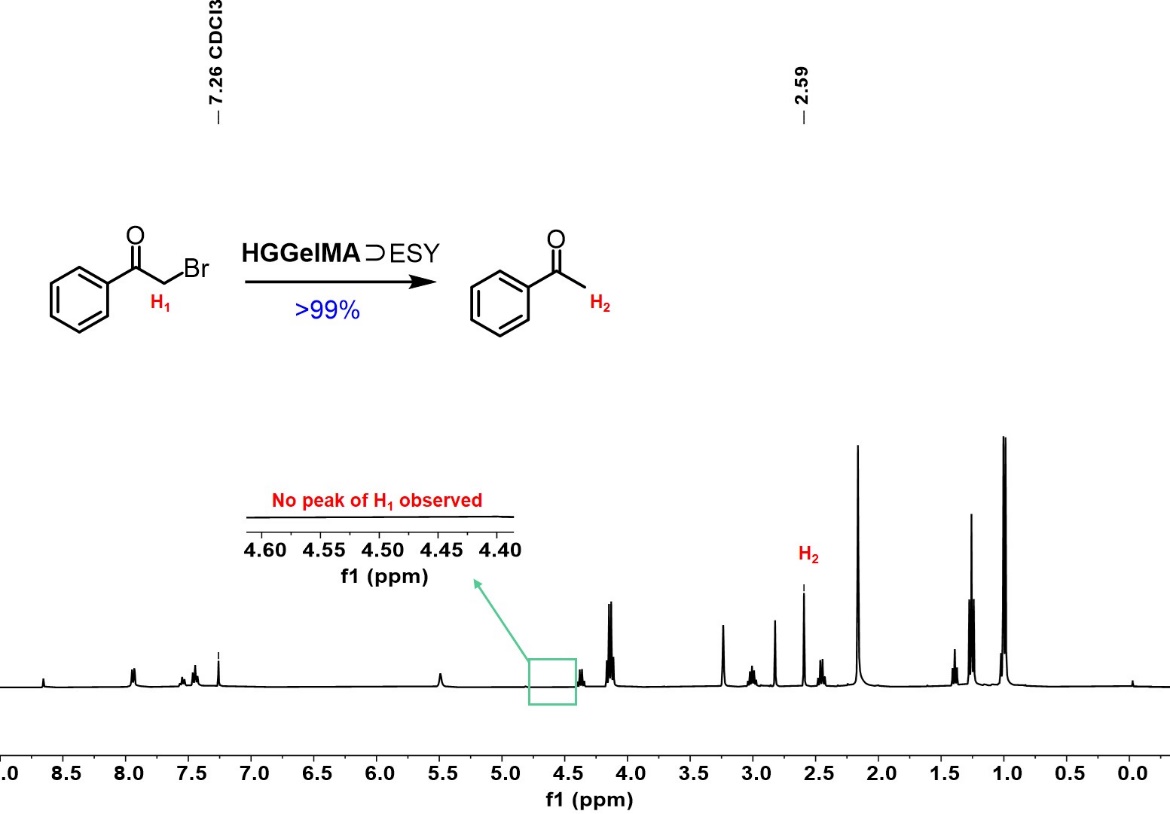


**Figure S16.** ^1^H NMR (400 MHz, CDCl_3_, 298 K) spectrum of the reaction mixture of entry 4.


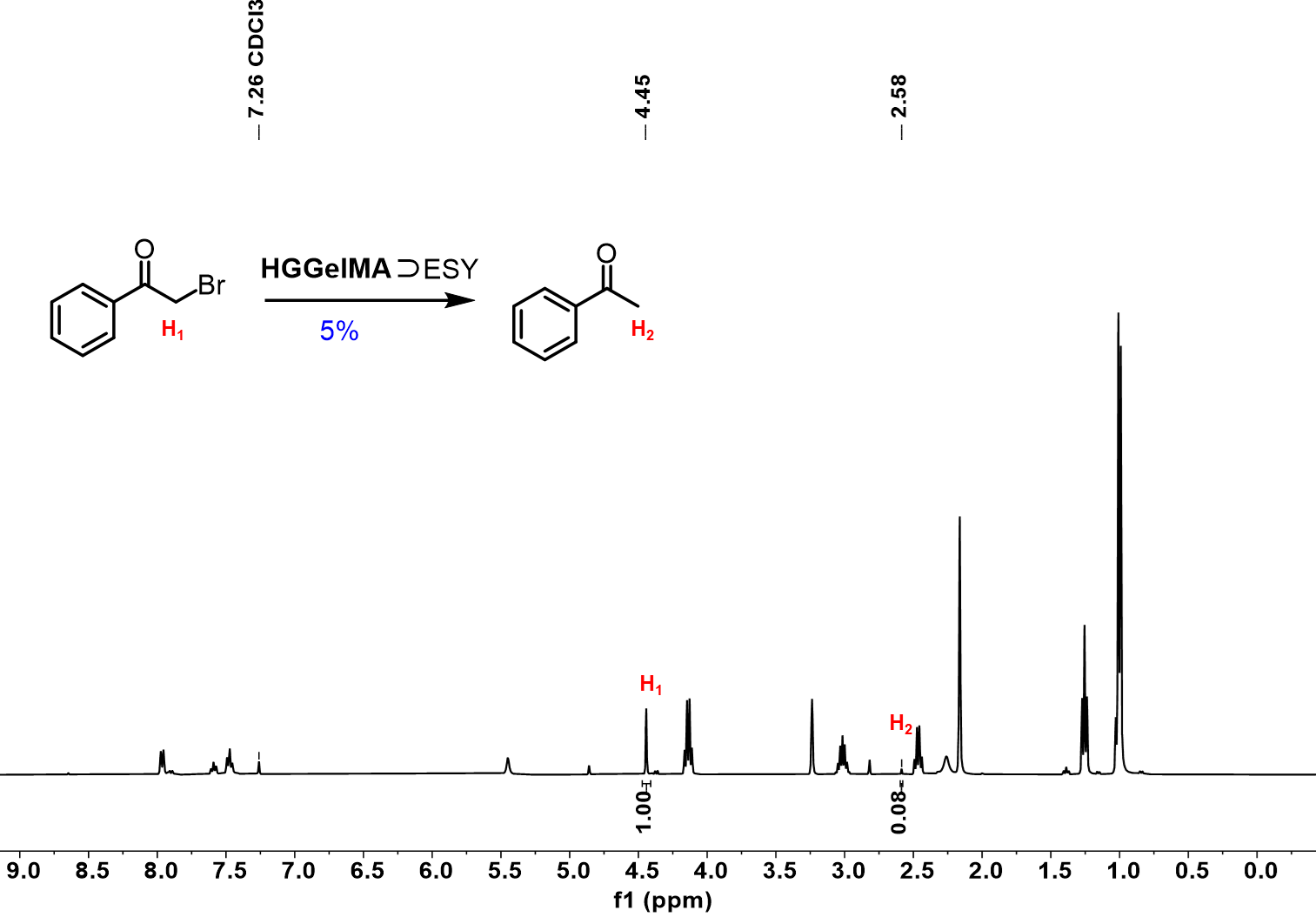


**Figure S17.** ^1^H NMR (400 MHz, CDCl_3_, 298 K) spectrum of the reaction mixture of entry 5.


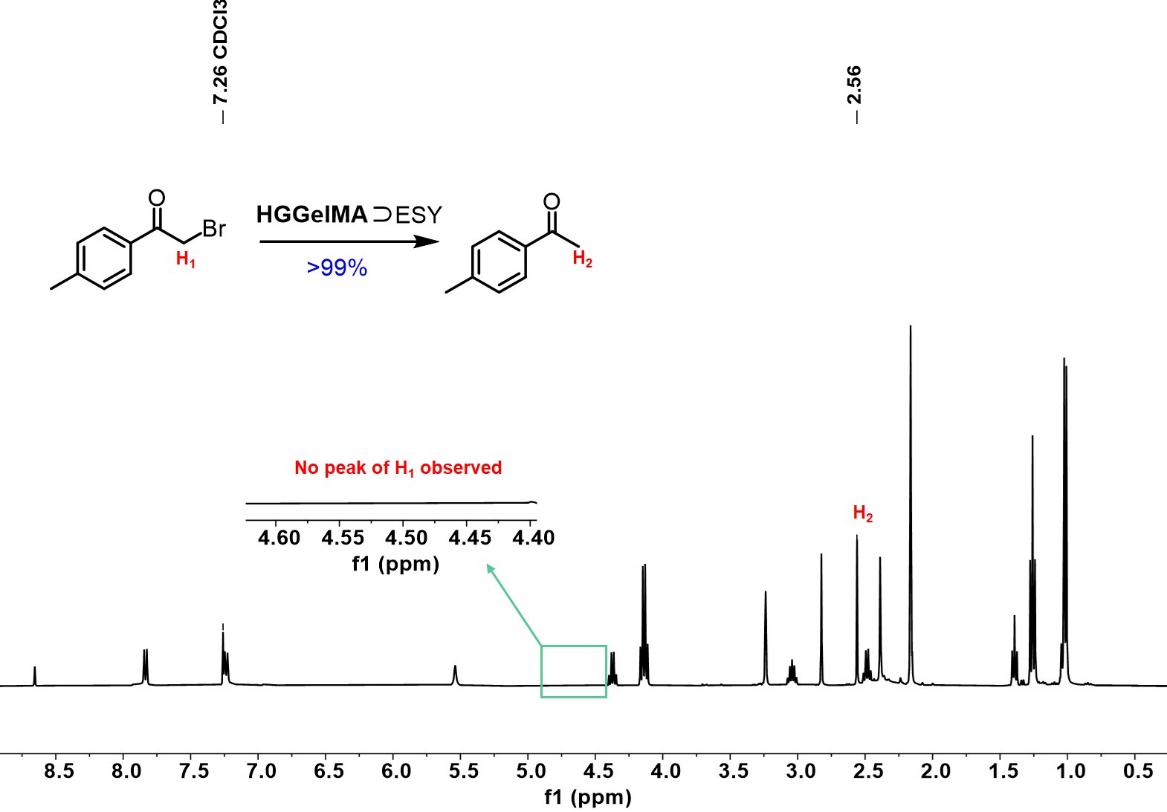


**Figure S18.** ^1^H NMR (400 MHz, CDCl_3_, 298 K) spectrum of the reaction mixture of entry 6.


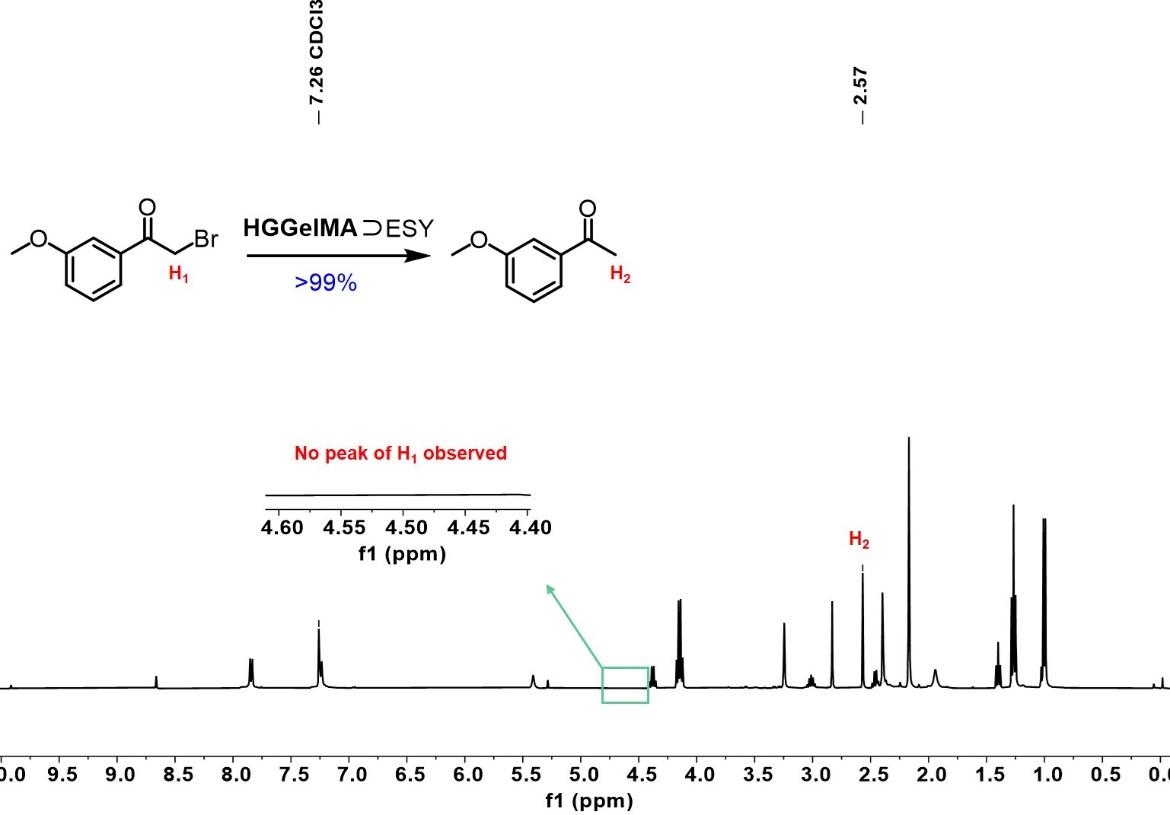


**Figure S19.** ^1^H NMR (400 MHz, CDCl_3_, 298 K) spectrum of the reaction mixture of entry 7.


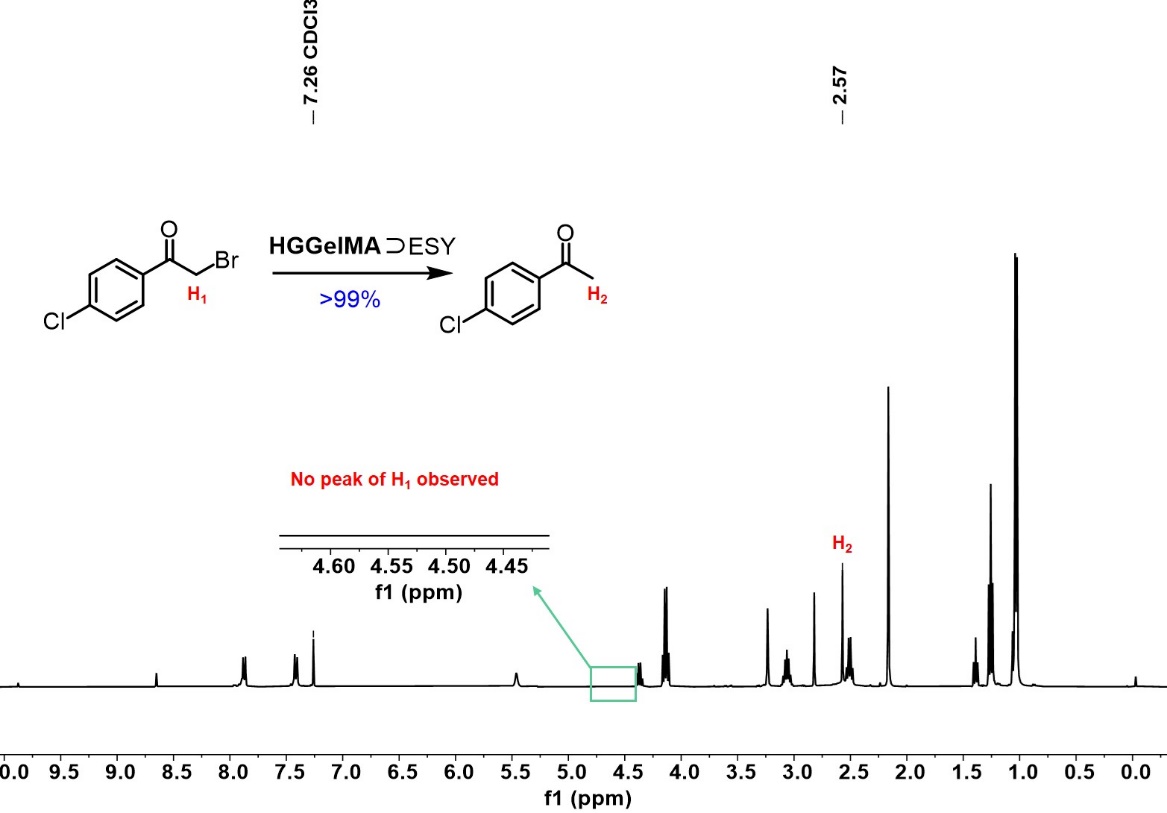


**Figure S20.** ^1^H NMR (400 MHz, CDCl_3_, 298 K) spectrum of the reaction mixture of entry 8.


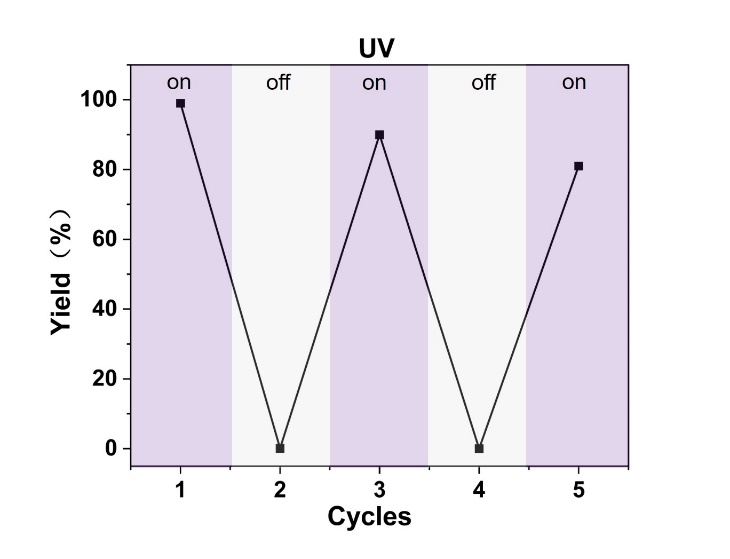


**Figure S21.** Yields after different cycles of the dehalogenation reaction using catalyst **HGGelMA**⊃ESY.

10. Proposed mechanism for the 2-bromo-1-phenylethanone dehalogenation reaction


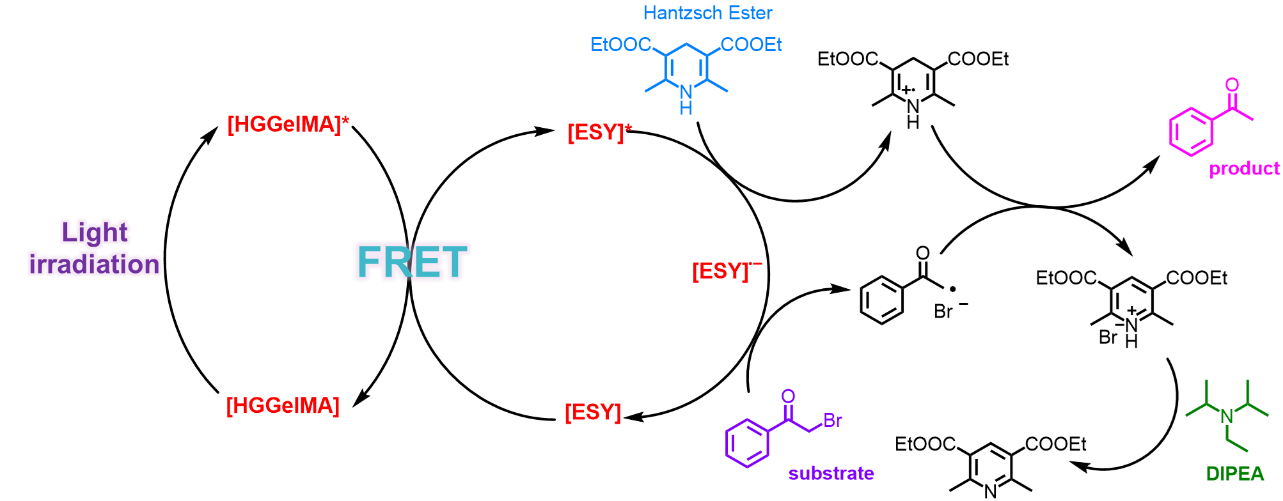


**Figure S22.** Proposed mechanism for the 2-bromo-1-phenylethanone dehalogenation reaction mediated by **HGGelMA**⊃ESY as a photocatalyst.

11. Investigation of ^1^O_2_ generation


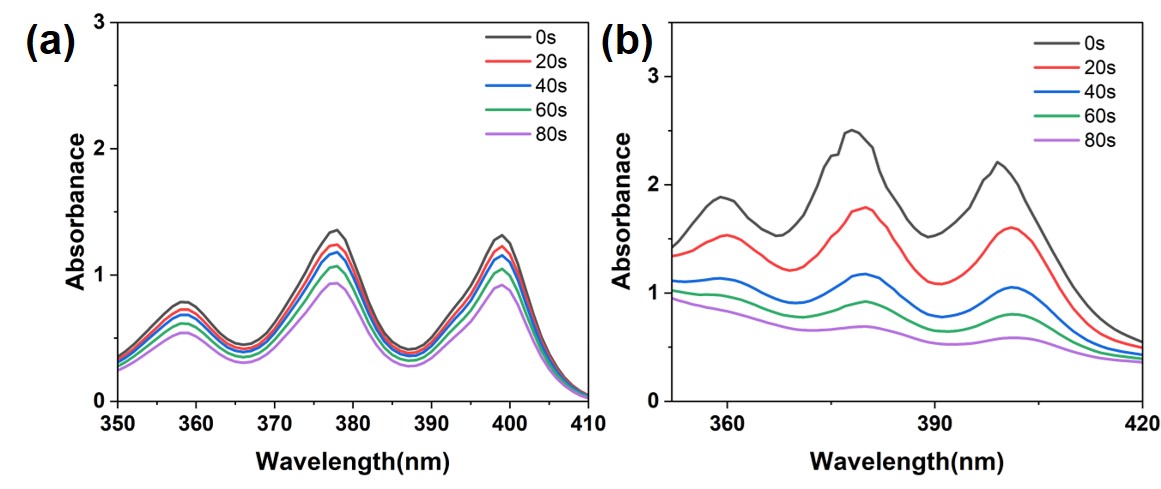


**Figure S23.** UV-Vis absorption spectra of ABDA after irradiation for varying durations (365 nm, 32 W): (a) Control: aqueous solution of ABDA without any additives; (b) aqueous solution of ABDA with the addition of **HGGelMA**⊃ESY.


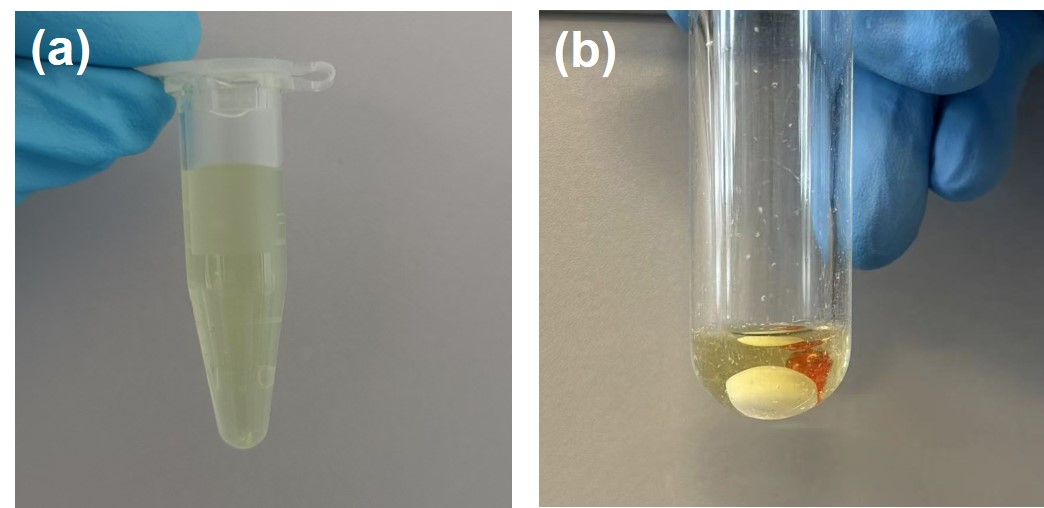


**Figure S24.** (a) Photograph of aqueous NBT solution; (b) Photograph of aqueous NBT solution after the addition of **HGGelMA**⊃ESY and subsequent irradiation with UV lamp (365 nm, 32 W) for 12 h under an oxygen atmosphere.

12. Investigation of oxidative coupling reactions

Table S3. Oxidative coupling reactions of benzylamine and its derivatives under different reaction conditions.

| Entry | R | Photocatalyst^[a]^ | Light irradiation | Yield^[b]^ |
| --- | --- | --- | --- | --- |
| 1 | H | None | Yes | <1% |
| 2 | H | ESY | Yes | 7% |
| 3 | H | **HGGelMA** | Yes | 10% |
| 4 | H | **HGGelMA**⊃ESY | Yes | 69% |
| 5 | H | **HGGelMA**⊃ESY | No | No reaction |
| 6 | *p*-Me | **HGGelMA**⊃ESY | Yes | 45% |
| 7 | *p*-OMe | **HGGelMA**⊃ESY | Yes | 61% |
| 8 | *p*-Cl | **HGGelMA**⊃ESY | Yes | 36% |

[a] Reaction conditions: benzylamine (0.02 mL, 0.18 mmol), acetonitrile (1 mL), **HGGelMA**⊃ESY (0.03% mmol), 32 W UV light, rt, O_2_, 12 h; [b] Product yield was obtained from ^1^H NMR spectra.

Since **HGGelMA**⊃ESY is in a solid state, CD_2_Cl_2_ was selected as the extraction solvent to isolate the product and substrate from the reaction solution after completion. In the ^1^H NMR spectrum, the signal peak of 1,3,5-trimethylbenzene was distinct and did not overlap with that of the product. Therefore, this compound was chosen as an internal standard and added to the reaction mixture. For calibration purposes, only -CH_2_ of the product and -CH on the benzene ring of the internal standard were selected.

The ratio between product and internal standard: r_A/Is_= (*B_1_*/*m_1_*)/(*B_2_*/*m_2_*), n_IS_=*m_IS_*/*M_IS_* can be determined by weighing a specific amount of internal standard. Subsequently, the following equation is used to calculate the exact data.

n_A_ = n_IS_⋅r_A/IS_

Finally, the yield of the product was obtained by using the following equation: *η*=n_A_/n_th_ % ^[7]^

Herein, *B_1_* represents the integral area of the -CH group in the product, *m_1_* denotes the number of protons corresponding to this characteristic peak. *B_2_* represents the integral area of the -CH_2_ group in the product, *m_2_* denotes the number of protons corresponding to this characteristic peak. *m_IS_* represent the weight of internal standard, *M_IS_* represents the relative molecular mass of internal standard, nth represents the theoretical yield of product *N*-phenylmethylene (0.09 mmol).


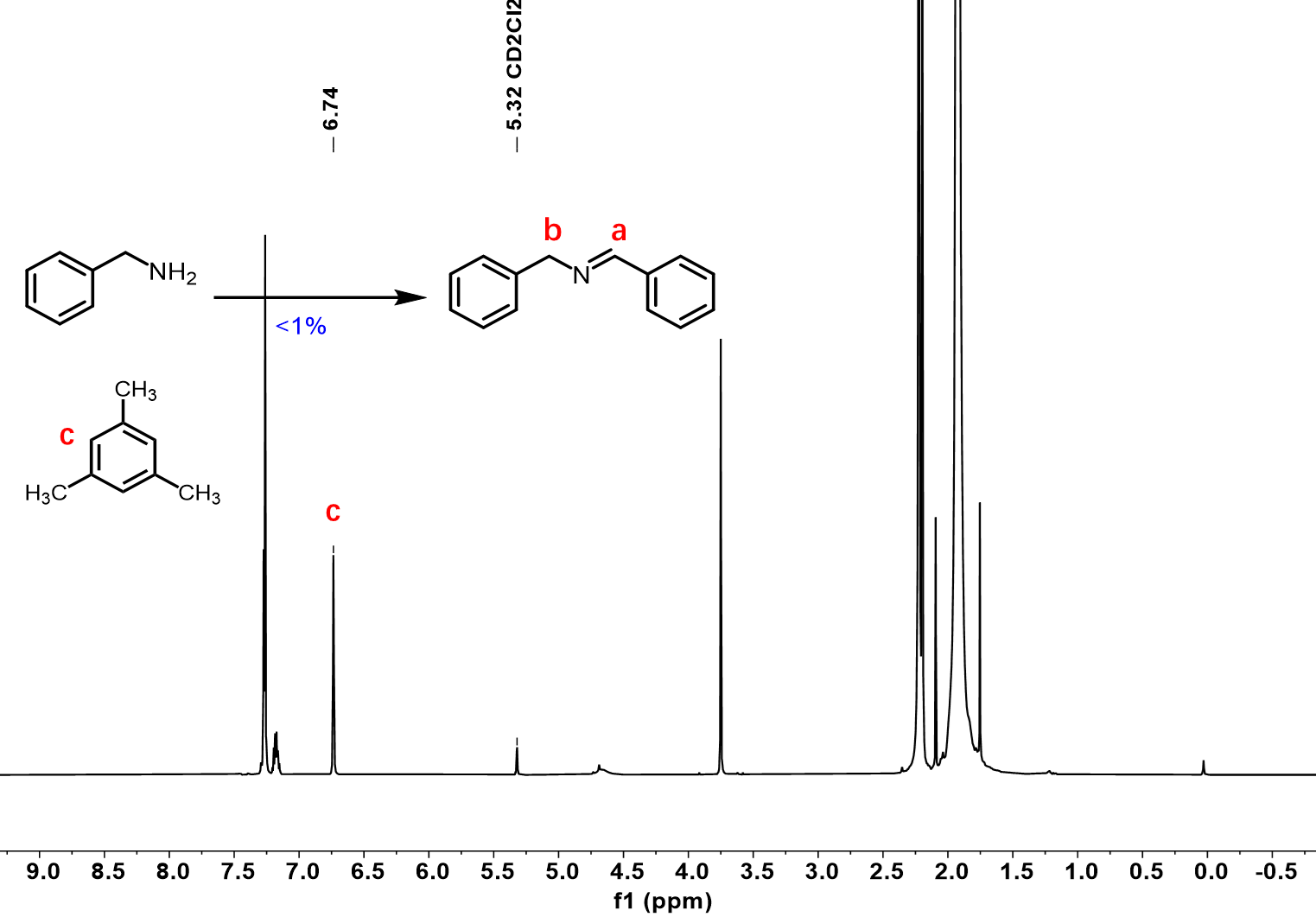


**Figure S25.** ^1^H NMR (400 MHz, CD_2_Cl_2_, 298 K) spectrum of the reaction mixture of entry 1.


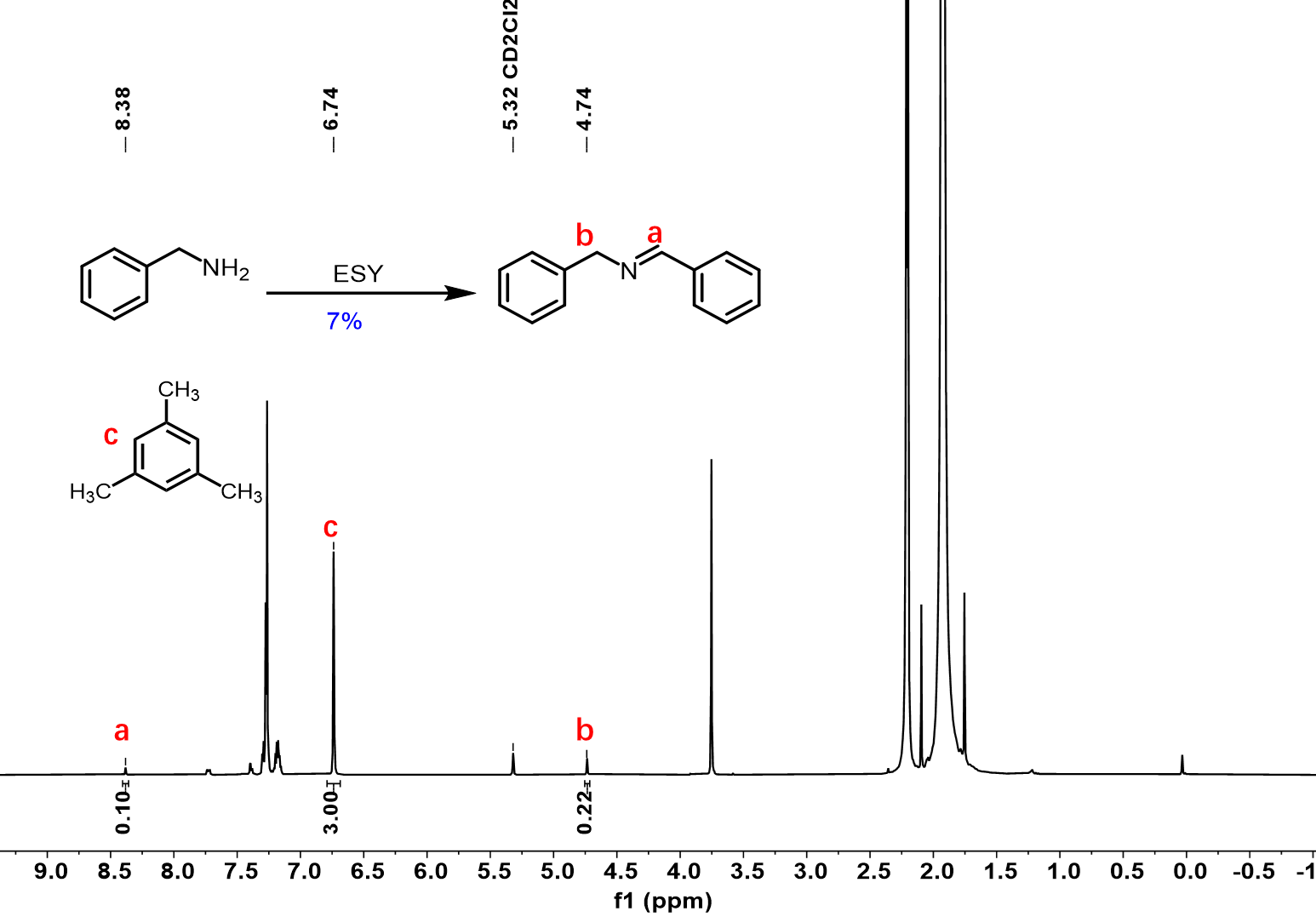


**Figure S26.** ^1^H NMR (400 MHz, CD_2_Cl_2_, 298 K) spectrum of the reaction mixture of entry 2.


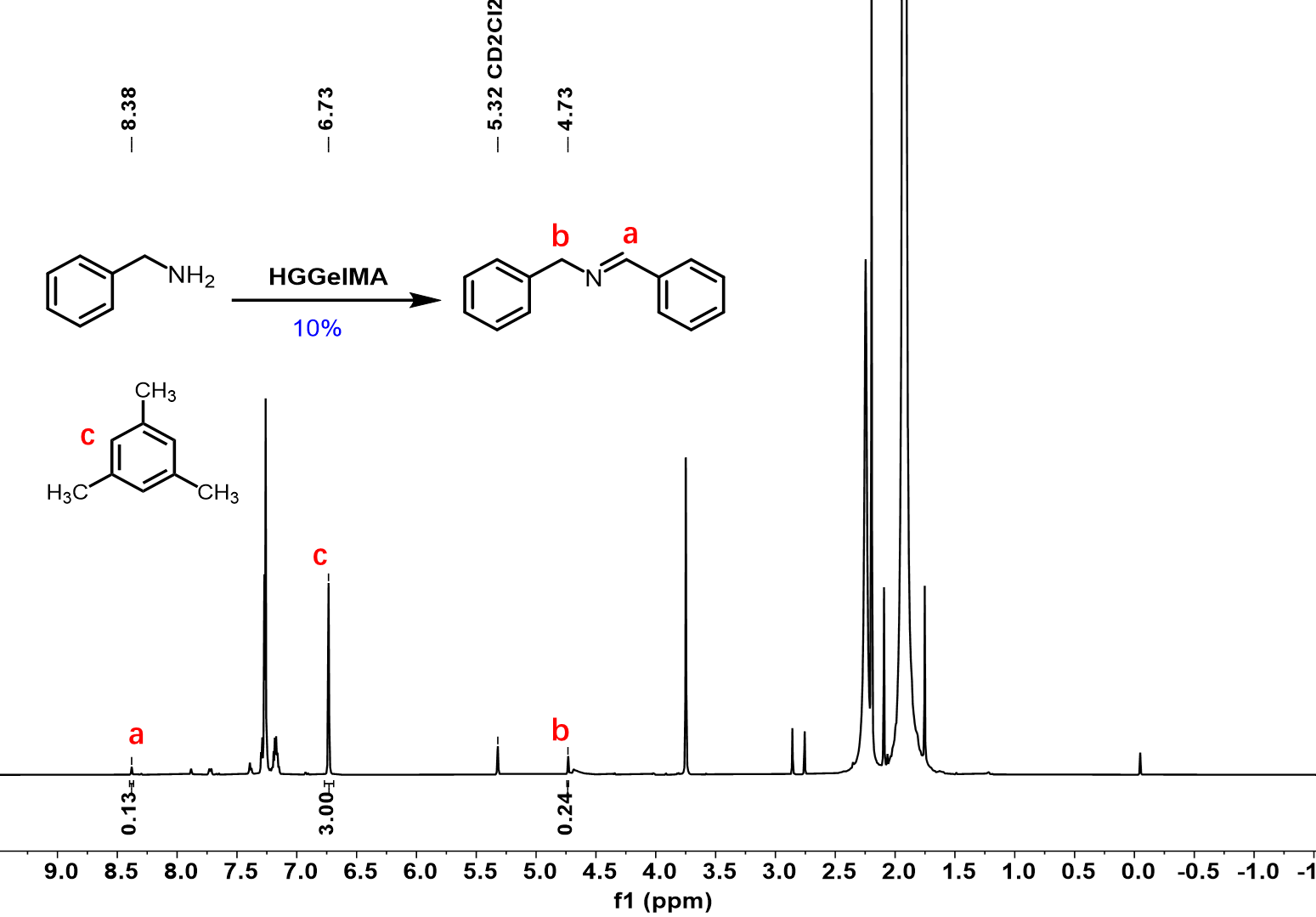


**Figure S27.** ^1^H NMR (400 MHz, CD_2_Cl_2_, 298 K) spectrum of the reaction mixture of entry 3.


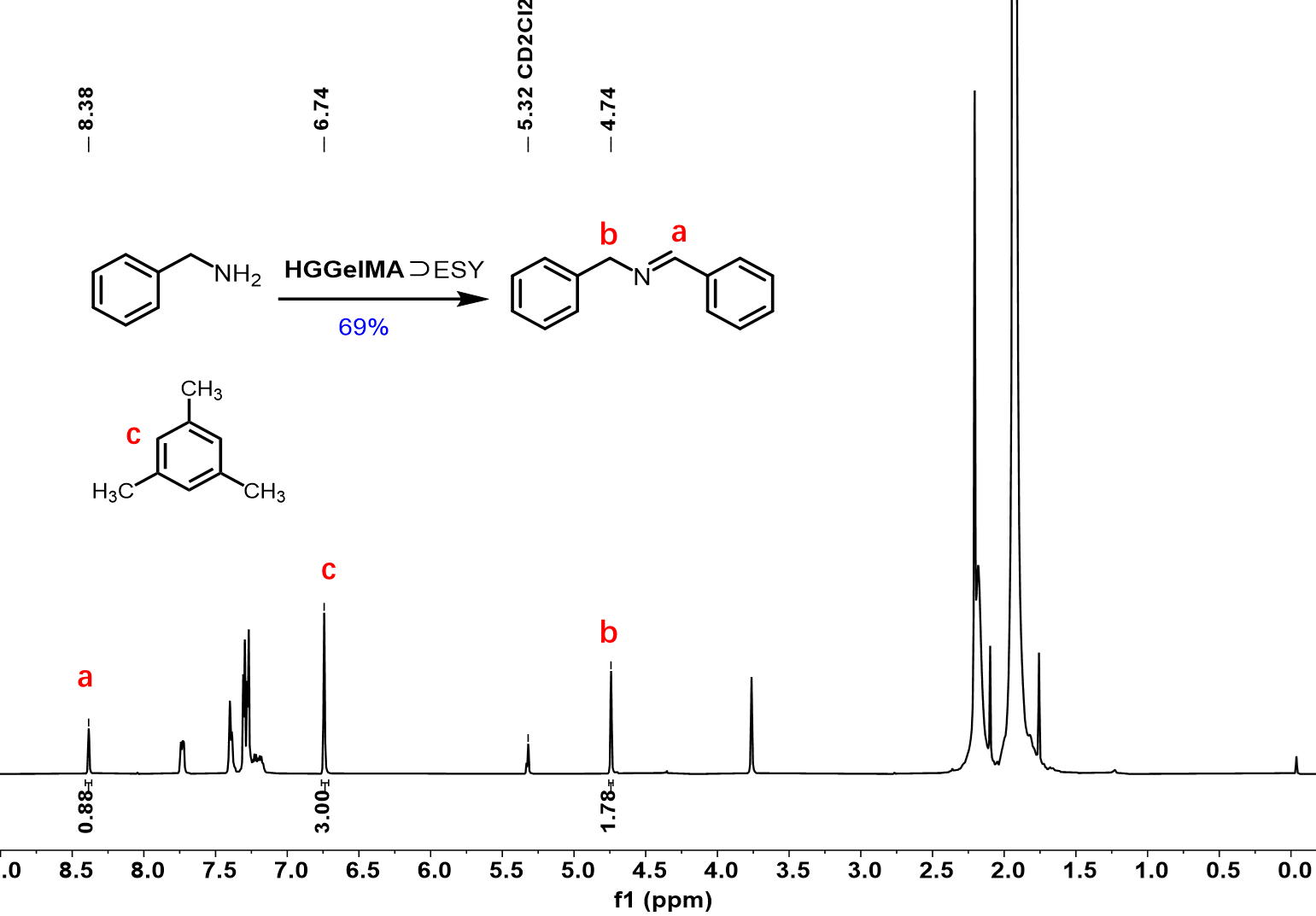


**Figure S28.** ^1^H NMR (400 MHz, CD_2_Cl_2_, 298 K) spectrum of the reaction mixture of entry 4.


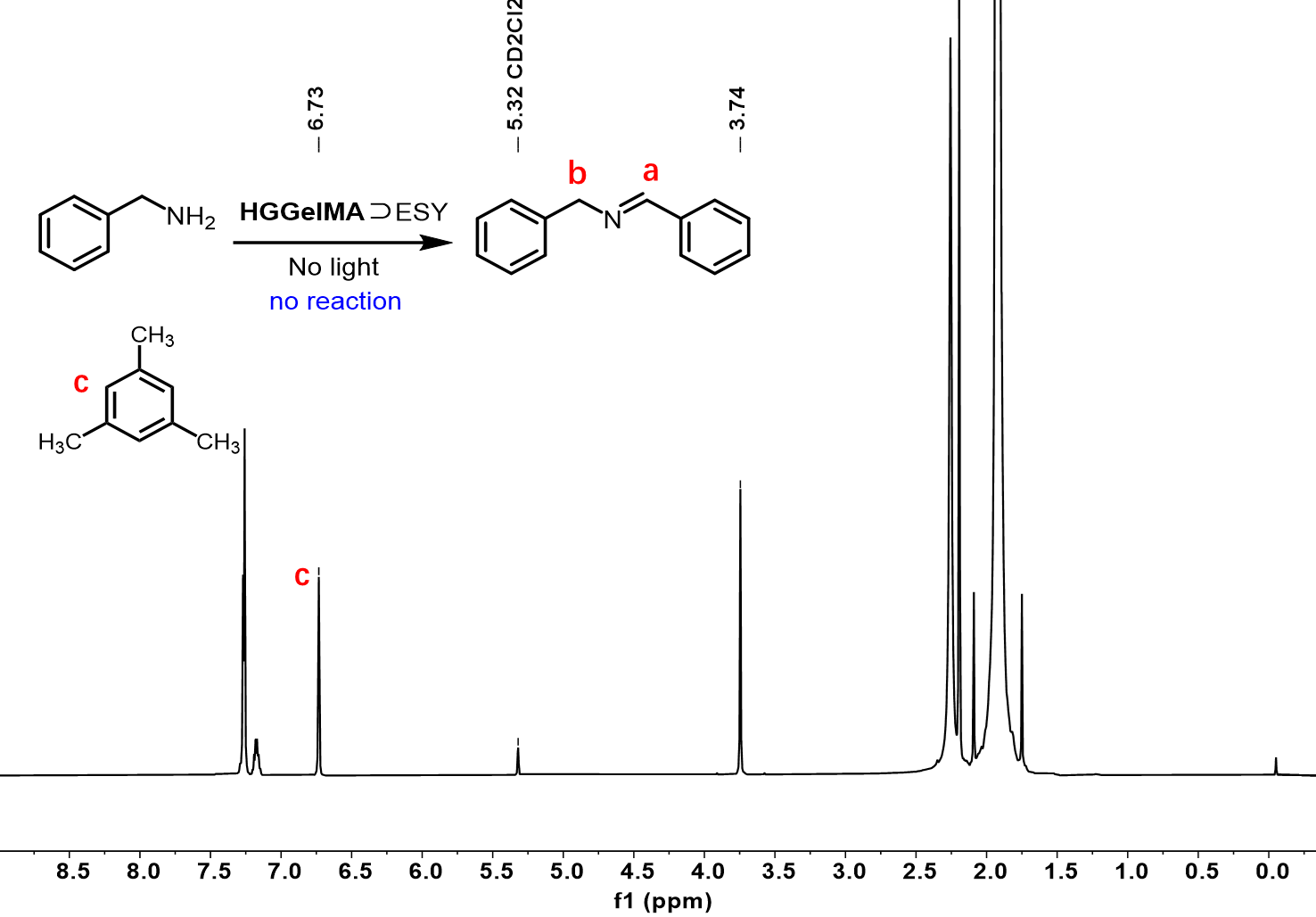


**Figure S29.** ^1^H NMR (400 MHz, CD_2_Cl_2_, 298 K) spectrum of the reaction mixture of entry 5.


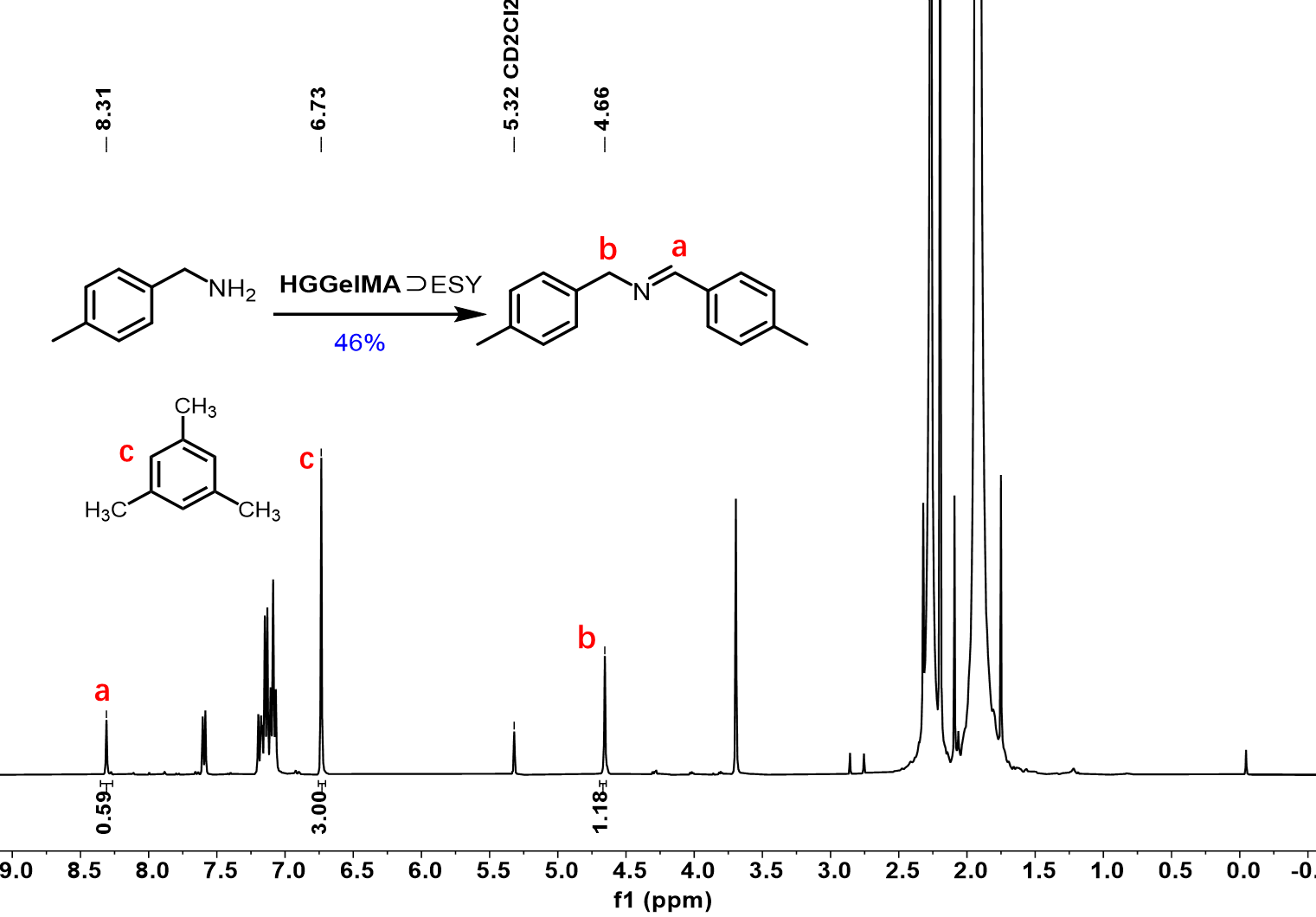


**Figure S30.** ^1^H NMR (400 MHz, CD_2_Cl_2_, 298 K) spectrum of the reaction mixture of entry 6.


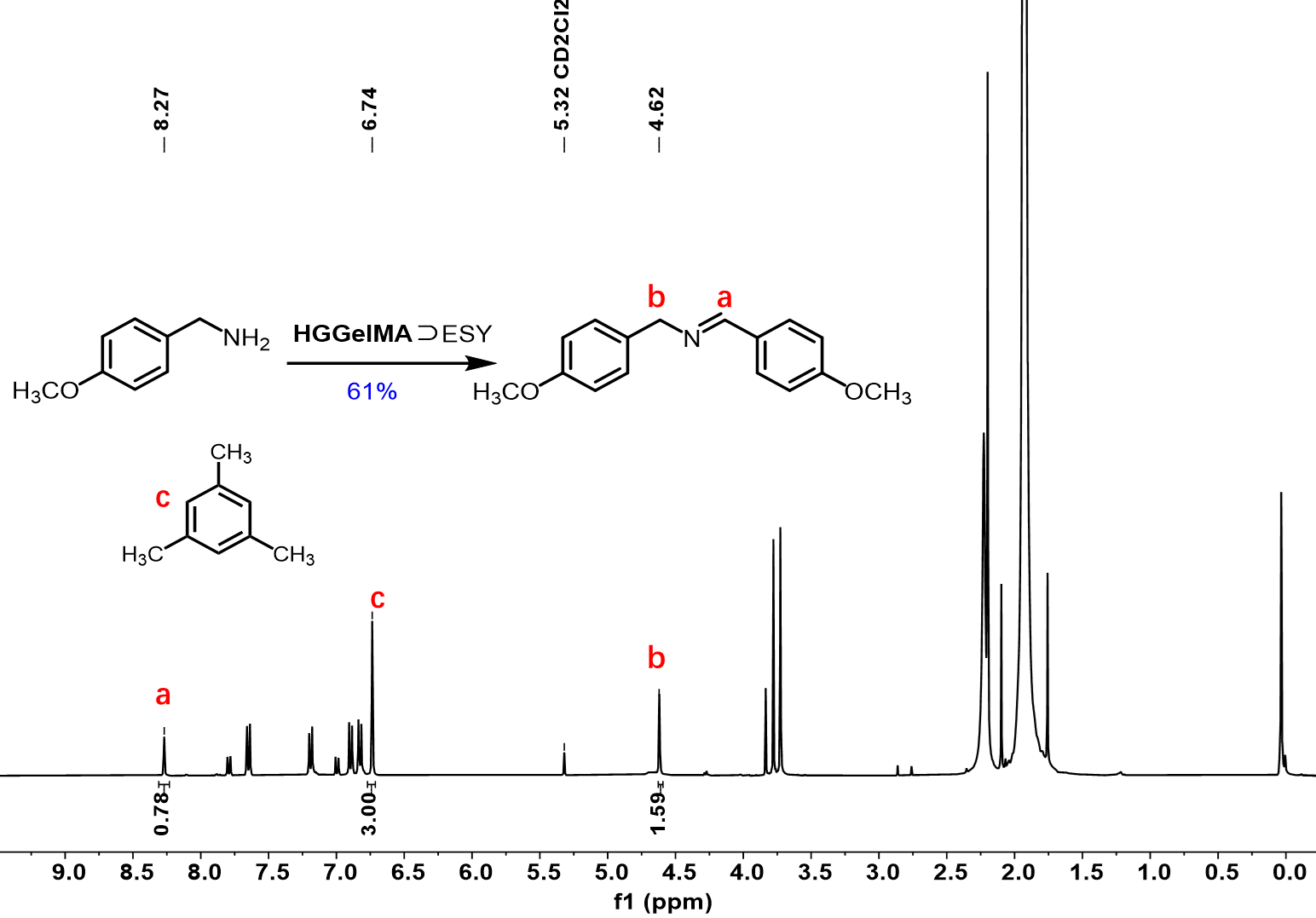


**Figure S31.** ^1^H NMR (400 MHz, CD_2_Cl_2_, 298 K) spectrum of the reaction mixture of entry 7.


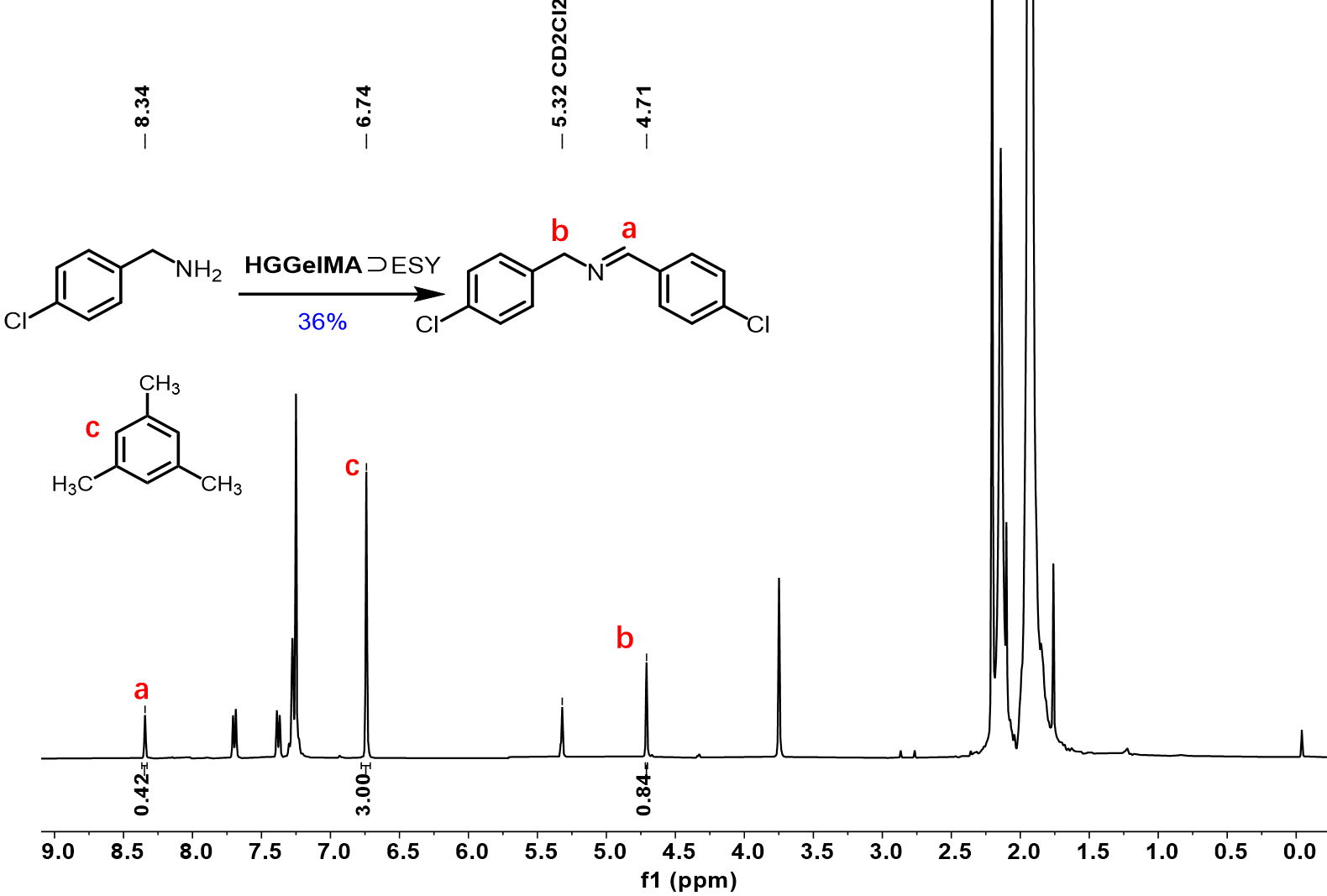


**Figure S32.** ^1^H NMR (400 MHz, CD_2_Cl_2_, 298 K) spectrum of the reaction mixture of entry 8.


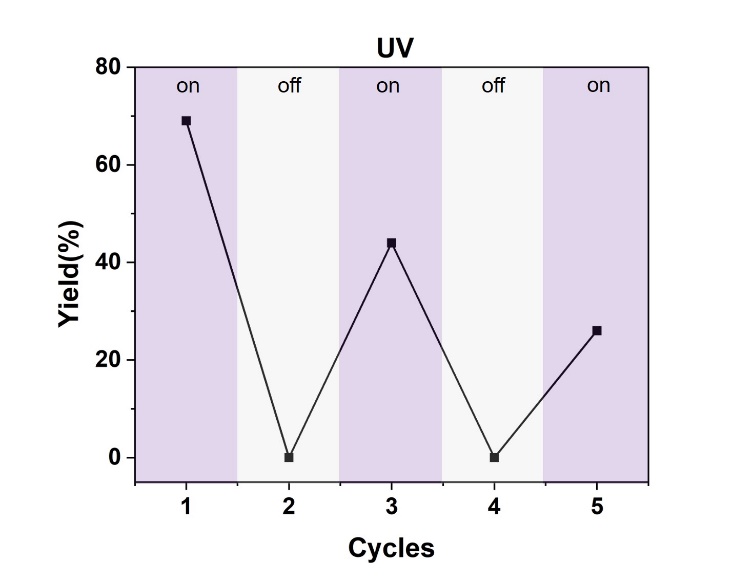


**Figure S33.** Yields after different cycles of the oxidative coupling reaction using catalyst **HGGelMA**⊃ESY.

10. Proposed reaction mechanism for the oxidative coupling of benzylamines


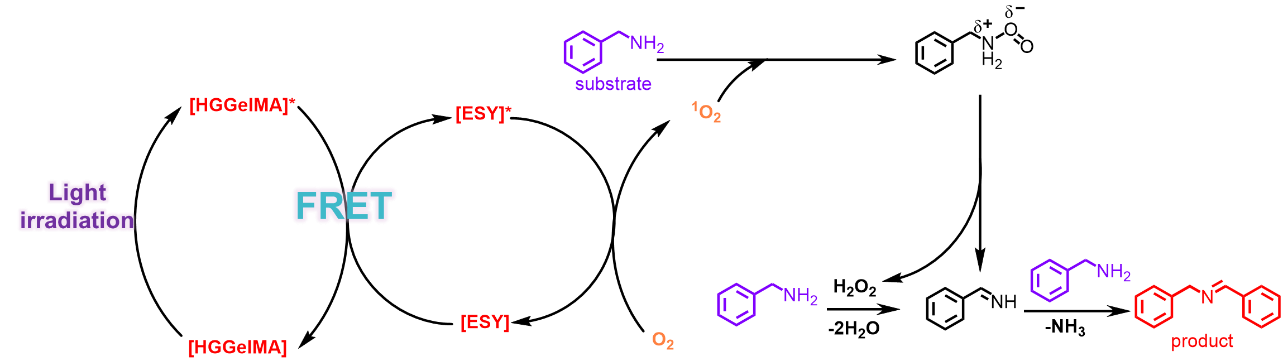


**Figure S34.** Proposed mechanism for the oxidative coupling reaction of benzylamine mediated by **HGGelMA**⊃ESY as a photocatalyst.

# 11. References

[1] X. Tian, M. Zuo, P. Niu, K. Velmurugan, K. Wang, Y. Zhao, L. Wang, X.-Y. Hu; Orthogonal Design of a Water-Soluble meso-Tetraphenylethene-Functionalized Pillar[5]arene with Aggregation-Induced Emission Property and Its Therapeutic Application. *ACS Appl. Mater. Interfaces* **2021**, *13*, 37466-37474.

[2] J.-F. Chen, P. Chen; Pillar[5]arene-Based Resilient Supramolecular Gel with Dual-Stimuli Responses and Self-Healing Properties. *ACS Appl. Polym. Mater.* **2019**, *1*, 2224-2229.

[3] Z. Wang, G. An, Y. Zhu, X. Liu, Y. Chen, H. Wu, Y. Wang, X. Shi, C. Mao; 3D-Printable Self-Healing and Mechanically Reinforced Hydrogels with Host-Guest Non-Covalent Interactions Integrated into Covalently Linked Networks. *Mater. Horiz.* **2019**, *6*, 733-742.

[4] J. J. Li, Y. Chen, J. Yu, N. Cheng, Y. Liu; A Supramolecular Artificial Light-Harvesting System with an Ultrahigh Antenna Effect. *Adv. Mater.* **2017**, *29*, 1701905.

[5] G. Sun, W. Qian, J. Jiao, T. Han, Y. Shi, X.-Y. Hu, L. Wang; A Highly Efficient Artificial Light-Harvesting System with Two-Step Sequential Energy Transfer Based on Supramolecular Self-Assembly. *J. Mater. Chem. A* **2020**, *8*, 9590-9596.

[6] K. Wang, R. Zhang, Z. Song, K. Zhang, X. Tian, S. Pangannaya, M. Zuo, X.-Y. Hu; Dimeric Pillar[5]arene as a Novel Fluorescent Host for Controllable Fabrication of Supramolecular Assemblies and Their Photocatalytic Applications. *Adv. Sci.* **2023**, *10*, 2206897.

[7] Z. Bai, K. Velmurugan, X. Tian, M. Zuo, K. Wang, X.-Y. Hu; Tetraphenylethylene-Embedded Pillar[5]arene-Based Orthogonal Self-Assembly for Efficient Photocatalysis in Water. *Beilstein J. Org. Chem.* **2022**, *18*, 429-437.

# Author Contributions

R. Zhang, X. Tian, and M. Zuo contributed equally to this work. R. Zhang, X. Tian, and M. Zuo drafted the manuscript and conceived the project. X.-Y. Hu supervised the project and revised the manuscript. R. Zhang and X. Tian performed the experiments. T. Zhang and S. Pangannaya revised the manuscript. All authors collectively analyzed the data, discussed the results, and provided comments on the manuscript.
